# Lightweight and ultrastrong 3D nanoarchitected high-entropy ceramic metamaterials

**DOI:** 10.1126/sciadv.adw6632

**Published:** 2025-10-17

**Authors:** Modong Jiang, Rui Li, Binzhao Li, Jincheng Ni, Guorui Wang, Zhong Zhang, Yang Chen, Yanlei Hu, Dong Wu, Jiaru Chu, Heng-An Wu, Jiawen Li

**Affiliations:** ^1^ CAS Key Laboratory of Mechanical Behavior and Design of Materials, Key Laboratory of Precision Scientific Instrumentation of Anhui Higher Education Institutes, Department of Precision Machinery and Precision Instrumentation, University of Science and Technology of China, Hefei 230027, China.; ^2^ CAS Key Laboratory of Mechanical Behavior and Design of Materials, Department of Modern Mechanics, University of Science and Technology of China, Hefei 230027, China; State Key Laboratory of Nonlinear Mechanics, Institute of Mechanics, Chinese Academy of Science, Beijing 100190, China.; ^3^ CAS Key Laboratory of Mechanical Behavior and Design of Materials, Department of Modern Mechanics, CAS Center for Excellence in Complex System Mechanics, University of Science and Technology of China, Hefei 230027, China.

## Abstract

Three-dimensional (3D) nanoarchitecture ceramics, such as ceramic nanolattices, have attracted intensive research interest due to good thermal stability, oxidation resistance, and damage tolerance. The high performance of lightweight ceramic nanolattices is still a goal to pursue. Herein, we report a high-entropy ceramic (HEC) 3D architecture with feature size down to 150 nanometers, exhibiting simultaneous high strength and energy absorption. A versatile strategy is proposed to synthesize fully transparent precursors with metal salt loading of up to 70%, which allows for high-resolution optical nanofabrication. Combining two-photon polymerization with a two-step sintering process, we fabricate fully dense and high-fidelity HEC 3D architectures. The high-entropy effect promotes the generation of high-density dislocations, thus enhancing both the strength and ductility of HEC nanolattices. This study demonstrates a promising strategy for developing exceptional-performance ceramics, with engineering application prospects in mechanical metamaterials, nanoelectromechanical systems, and damage-tolerant lightweight materials.

## Teaser

A universal strategy for fabricating nanoarchitected ceramics achieved 3D HEC nanolattice with high strength and ductility.

## INTRODUCTION

Mechanical metamaterial is a type of structural materials that obtain special properties beyond that of natural conventional materials through artificially designed micro/nanoscale geometries. Propelled by the rapid development of nanoscale three-dimensional (3D) printing techniques (*1*–*4*), nanolattices with optimized topologies have demonstrated a particularly promising pathway to achieve the combination of mechanical properties that are generally mutually exclusive, such as ultrahigh strength-to-density ratios (*1*, *5*, *6*). Several strategies exist for precisely crafting such nanomaterials, most of which use lasers to induce patterned “photopolymerization” of light-sensitive materials. In addition, over the past few years, scientists have made considerable headway in overcoming the limitations that have impeded broader adoption of these methods (*2*, *7**–**9*). However, one of the remaining challenges is that not all materials can be printed directly through photopolymerization, such as ceramics. Compared to polymers and alloys, ceramics are the ideal material for nanolattices working in high-stress and extremely harsh environments due to their high-temperature stability, oxidation resistance, and damage tolerance (*10*, *11*). Hence, the precise fabrication of 3D architected ceramics at the nanoscale is expected to push the attainable performance and applications of structural materials to unprecedented regions.

So far, most works on ceramic nanolattices have been focused on nanostructures with hollow-beams (with the template removed) or polymer-core ceramic-shell beams fabricated through the atomic layer deposition on polymer templates (*12**–**14*). The strength of these lightweight ceramic nanolattices is significantly reduced because of pipe wall fracture or the poor strength of the polymer core. Recently, ceramic resins compatible with high-resolution 3D lithography technology, like two-photon polymerization (2PP), have been developed to fabricate 3D nanoarchitected solid ceramics (*3*, *15**–**22*). Despite the great progress, some issues still have not been well resolved, such as the complex procedure of feedstock preparation, limited applicable materials, poor optical transparency, and low ceramic constituent loading. These problems have restricted the stable and repeatable fabrication of fully dense and high–shape fidelity ceramic nanolattices with a feature size down to 150 nm and have limited the performance breakthrough of ceramic nanolattices by the size effects (*23*). Moreover, to best of our knowledge, there are few reports on multicomponent solid solution ceramics, also named as high-entropy ceramic (HEC), with nanoscale 3D frameworks, which can explore potential enhancement of high-entropy effect on mechanical metamaterial.

Here, a simple and versatile synthesis method using acid-base neutralization reactions has been developed to prepare more than 15 fully transparent photosensitive metal acrylates without any organic solvents or cross-linkers. A metal acrylate loading of up to 70% is achieved without affecting the optical transparency, which allows for the subsequent lithography nanofabrication. Based on the proposed method, a HEC photosensitive resin has been prepared to manufacture HEC nanolattices via 2PP direct laser writing (2PP-DLW) and two-step sintering. To obtain fully dense HEC nanolattices with high shape fidelity, the evolution of grain size and densification during sintering process as well as the influence of anchoring effect on structural shrinkage have been comprehensively studied. As a result, the feature size of HEC nanolattices has been reduced to 150 nm. Benefiting from high-density dislocations generated by high entropy, the HEC micropillar with a pre–failure strain of more than 10% exhibits high compressive strength far superior to low-entropy and medium-entropy compositions. Further experiments find that the lightweight shell-based HEC nanolattices exhibit the simultaneous ultrahigh specific strength of more than 1 GPa g^−1^ cm^3^ and energy absorption of up to 230 MJ m^−3^ with a maximum fracture strain of 0.4, which are even comparable to state-of-the-art pyrolytic carbon (PC) nanolattices. Additional finite element (FE) simulation and dynamic mechanical analysis (DMA) further confirm the HEC enhanced toughness in 3D structures, which has enormous potential in many emerging engineering applications. Overall, this work has overcome various limitations that have long existed in the preparation of ceramic resins and provided a previously unexplored way to further explore the excellent performance of multicomponent ceramic materials with 3D architectures at nanoscale.

## RESULTS

### Fabrication of HEC 3D nanolattices

**Fig. 1.**
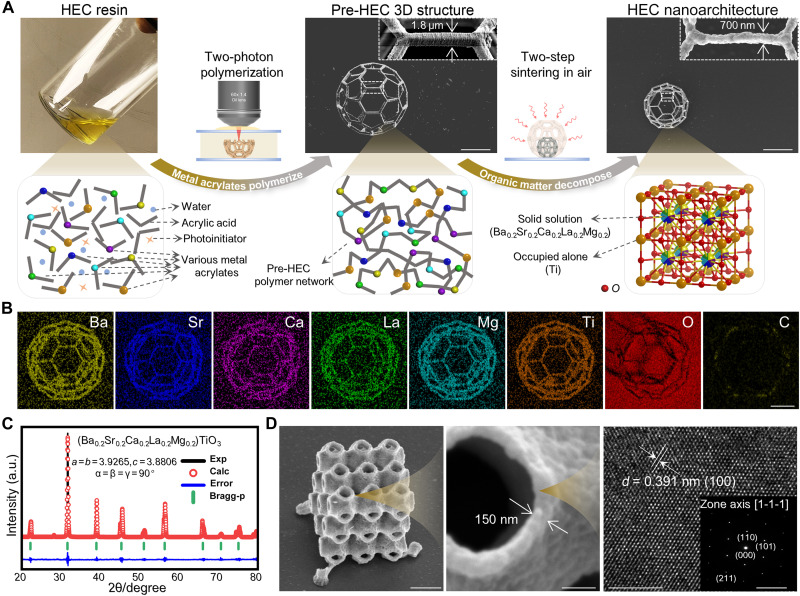
Preparation of high-resolution HEC nanolattices from metal acrylates precursors. (**A**) Schematic synthesis HEC nanoarchitectures through 2PP-DLW and subsequent two-step sintering in air. Scale bars, 20 μm. (**B**) Energy-dispersive spectroscopy (EDS) element distribution maps of C60 polyhedron–shaped HEC. Scale bars, 10 μm. (**C**) Rietveld refinement x-ray diffraction (XRD) pattern of the (Ba_0.2_Sr_0.2_Ca_0.2_La_0.2_Mg_0.2_)TiO_3_ HEC, revealing a P4mm crystalline phase. (**D**) A representative HEC Schwarz P nanolattice with a wall thickness of 150 nm. Transmission electron microscopy (TEM) and selected-area electron diffraction (SAED) both confirm the perovskite-type lattice of the tetragonal phase. Scale bars are 2 μm, 300 nm, 5 nm, and 5 nm^−1^, respectively (from left to right).

HEC is the solid solution of inorganic compounds with one or more lattice sites shared by equal or near-equal atomic ratios of multiprincipal elements (*24*). In this work, a HEC photosensitive resin mainly consisting of various fully transparent metal acrylates has been synthesized (Fig. 1A). Then, 2PP-DLW, which is a powerful technique to print 3D structures at nanoscale, is adapted here to print 3D preceramic polymer. After printing, the polymer scaffold structure is then subjected to two-step sintering in air to obtain the HEC nanolattices by ~50% linear shrinkage for each dimension.

Chemical composition of the as-fabricated HEC architectures has been characterized comprehensively. The energy-dispersive spectroscopy (EDS) mapping images of a C60 nanolattice indicate that all elements display a homogeneous distribution in HEC (Fig. 1B). In addition, the corresponding EDS spectrum (fig. S1) confirms that five metal elements occupy one lattice site by near-equal atomic ratios. In Fig. 1C, the Rietveld refinement of the x-ray diffraction (XRD) patterns indicates that the HEC exhibits a P4mm single phase. The surface chemical composition and electronic effects are further characterized by x-ray photoelectron spectroscopy (XPS). The XPS survey spectra validate the occurrence of the targeted metal elements in the designed HEC samples (fig. S2).

Based on the proposed strategy, the highest resolution of HEC 3D nanolattices with high shape fidelity has reached to 150 nm, as shown in Fig. 1D. Both the transmission electron microscopy (TEM) image and the corresponding selected-area electron diffraction (SAED) pattern display a clear tetragonal perovskite lattice with a clear lattice spacing of (100) measured as 0.391 nm.

### Composition design and resin synthesis of HEC

**Fig. 2.**
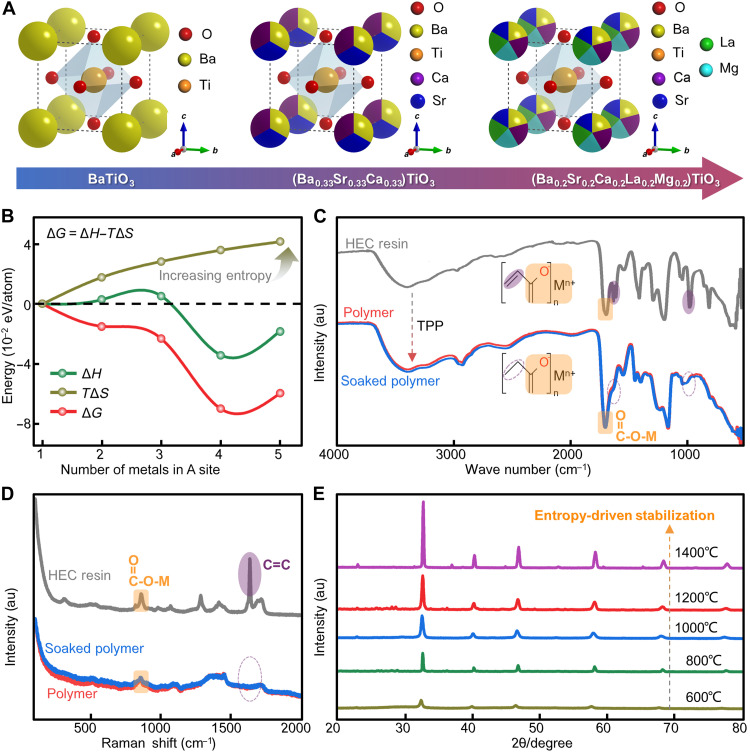
HEC constituent design and formation. (**A**) Schematic diagram of Sr, Ca, La, and Mg equiprobably replacing Ba step by step at position A to obtain disordered perovskite lattices with entropy increasing. (**B**) The Gibbs free energy (∆*G*), enthalpy (∆*H*), and entropy (∆*S*) vary as functions of the number of metals in A site. ATR-FTIR spectroscopy (**C**) and Raman spectroscopy (**D**) of HEC resin, polymer, and soaked polymer. (**E**) XRD patterns of HEC sintered under different temperatures. au, arbitrary units.

To ensure the formation of a solid solution ceramic from multimetal acrylates by 2PP-DLW and pyrolysis, a stable high-entropy structure with at least five compatible metal elements in one lattice site must be well designed and prepared (Fig. 2A and fig. S3) (*25**–**27*). Generally speaking, the stability of multicomponent systems is measured by size differences in lattice and thermodynamical energy changes. In this work, configuration entropy (Δ*S*) (*28*), Gibbs free energy (Δ*G*) (*29*), Schmidt tolerance factor (*t*_effective_) (*30*), and lattice size difference (δ) (*31*) have been taken to design a perovskite-type (ABO_3_) HEC (Supplementary Text).

Δ*S* is originating from disorder in crystals (*28*) and enhances when more metal elements are added to the ABO_3_ system (Fig. 2B and table S1). From the perspective of energy, an Δ*S* value ≥ 1.5R is regarded as high entropy. The constructed HEC with equal molar fractions of five elements in A site will maximize the Δ*S* value to be 1.69R, more than 1.5R. To ensure a stable phase thermodynamically, the Δ*G* of prepared HEC is suggested to be negative, whereas a positive value indicates thermodynamic phase separation (*29*). Notably, Δ*G* directly depends on the mixing enthalpy (Δ*H*), and Δ*S* values. Through the Vienna ab-initio simulation package (VASP) software, we successfully simulate the single-phase multicomponent ceramics with increasing metal elements in A site and obtain corresponding Δ*H* (figs. S4 and S5). Due to a high entropy, the designed HEC will generate a very negative Δ*G*, which is described as an entropy-driven phase stabilization effect (*28*). When introducing various metal atoms with different sizes into one lattice site, the targeted HEC lattice must undergo controllable contraction or elongation to maintain a single phase and exist stably (*32*). To design HEC with a perovskite phase, *t*_effective_ is a widely used criterion for determining whether a multicomponent system can form a stable crystal phase (*30*). It measures the deviation of lattice consisting of ions with different radius from the standard cubic phase, of which the value is 1. As shown in fig. S6 and table S1, the perovskite ceramics and HEC can exist stably because *t*_effective_ ranges from 0.8 to 1. Additionally, δ has been also used to measure the effect of the atomic-size difference on the structural stability (*31*). In brief, although the significantly distorted lattice from the unbalance among ionic size, mass, and bond state, a single-phase stable HEC (Ba_0.2_Sr_0.2_Ca_0.2_La_0.2_Mg_0.2_)TiO_3_ has been successfully designed by five compatible metal elements in A site of ABO_3_ due to the entropy-dominated phase stabilization effect and a negative ∆*G* (table S1).

To fabricate the designed HEC 3D nanostructure by high-resolution 2PP-DLW, an efficient and versatile method, which is capable to synthesize multiple transparent ceramic precursors, needs to be proposed. Here, the acid-base neutralization reaction between metal oxides or hydroxides and acrylic acid is used to yield metal acrylates (fig. S7A), which have been proven to be suitable for photopolymerization due to their carbon-carbon double bonds (*19*, *33*, *34*). Previous works have demonstrated that transition group metal alkoxides can undergo coordination-condensation reactions with carboxylic acids, forming metal-oxo clusters encapsulated by carboxylate ligands and improving their solubility in water (*35*). Building on this knowledge, we obtain highly transparent metal acrylates by adding a small amount of acrylic acid and water, requiring no organic solvents. Without complex synthesis steps, the highly transparent HEC resin with no visible particles or precipitates can be prepared directly by mixing corresponding metal acrylates and photoinitiator 7-(diethylamino)-3-phenylcoumarin (DETC), as shown in Fig. 1A. Based the attenuated total refraction (ATR)–Fourier transform infrared (FTIR) spectroscopy and Raman spectroscopy, DETC in the exposure area can generate a large number of free radicals through two-photon absorption during the 2PP-DLW process, which, in turn, triggers the polymerization of the carbon-carbon double bonds in the HEC resin (Fig. 2, C and D). Meanwhile, the overlapping characteristic peaks observed in polymer and soaked polymer validate that metal ions will not be washed out during the development process because they are bound to the polymer network through coordination bonds with carboxylate ligands. Moreover, different valence metal acrylates with abundant unsaturated carbon bonds can significantly promote cross-linking density of the polymer network without additional cross-linkers (fig. S7B). As a result, the HEC resin is almost entirely composed of metal acrylates (wt % of up to 70%), which contain metal elements of up to 13.5% (table S2). It is worth mentioning that our method for preparing HEC resin can be further expanded to configure more than 15 fully transparent metal acrylates precursors (fig. S8), thus fabricating various 3D ceramic nanoarchitectures with customizable constituents (fig. S9).

After printing, the organic compositions are removed by thermal annealing at high temperatures in air. With the temperature increased to 550°C, metal acrylates decompose completely, and the metal ions form various metal oxides owing to oxidation (fig. S10). To reveal the formation of HEC during sintering, XRD of HEC samples sintered under different temperatures is analyzed (Fig. 2E). All diffraction peaks matched those of standard P4mm single phase with no additional peaks observed. From 600° to 1400°C, the HEC transforms to high-purity polycrystalline ceramic. The narrowing of the full width at half maximum with temperature also indicates enhanced crystallinity of the HEC. This further demonstrates the thermodynamic stability of HEC crystal phase at different temperatures (fig. S11).

### Fully dense HEC 3D nanoarchitecture with high fidelity

**Fig. 3.**
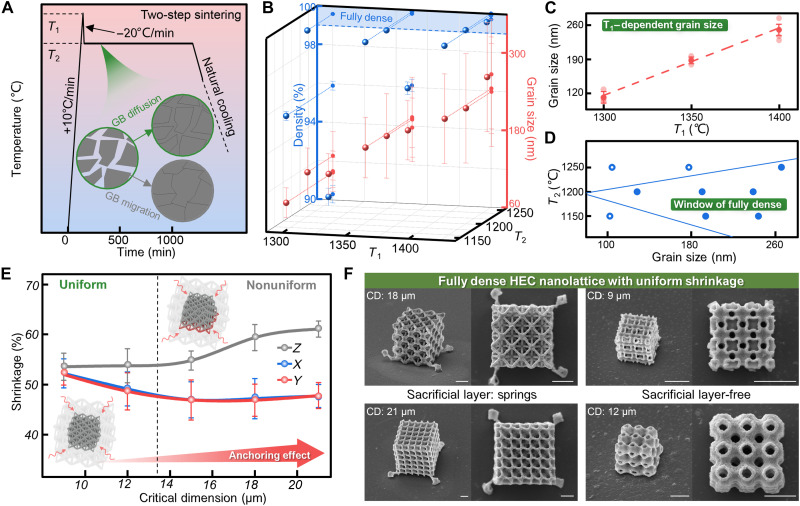
Fully dense HEC nanolattice with high fidelity. (**A**) Two-step sintering process for HEC. The inset displays grain-boundary (GB) diffusion and grain-boundary migration. (**B**) Grain size and density 3D distribution map under varying *T*_1_ and *T*_2_. A density of more than 99% is considered as fully dense due to statistical errors. (**C**) The grain size varies as function of *T*_1_. (**D**) A kinetic window for reaching full density without grain growth. (**E**) Shrinkage rates in the *x*, *y*, and *z* directions vary as functions of critical dimension. The inset shows anchoring effect during sintering. (**F**) Fully dense HEC 3D nanoarchitectures with uniform shrinkage. Scale bars, 3 μm. Data are presented as means ± SD. Data in [(B), (C), and (E)] are from *n* = 3 independent samples. CD, critical dimension.

In general, the final-stage sintering process of ceramic preparation is always accompanied by undesired coarse grains, which greatly hinders the production of functional ceramics with high-resolution nanoscale structures (*19*, *33*, *36*). To overcome this challenge, we adopt two-step sintering approach for HEC preparation (Fig. 3A). To succeed in two-step sintering, a sufficiently high starting density should be obtained in *T*_1_. Once cooled down to *T*_2_, the densification without grain growth can be achieved by the suppression of grain-boundary migration while keeping grain-boundary diffusion active (*37*). To further understand the underlying principles of two-step sintering, we systematically investigate the effect of sintering conditions on HEC grain and density evolution (Fig. 3B). As shown in Fig. 3C and fig. S12, it is observed that the grain size is basically dependent on *T*_1_ in the first step sintering. Figure 3D shows a kinetic “window” for fully dense HEC with different grain sizes. Smaller grain size means a narrower *T*_2_ range to achieve fully dense without grain growth in the second step sintering (fig. S13). This suggests that grain-boundary migration may have a higher activation energy than grain-boundary diffusion, which means that it is only active at a higher temperature. Meantime, it is possible that the activation process of grain-boundary migration and grain-boundary diffusion are both dependent on the size of existed grain boundary, and larger grain boundary is more benefit for atoms to move along grain boundary to fill grain gap instead of boundary movement with grain coarsening. This explains why the upper temperature in Fig. 3D shifts to a higher value at a larger grain size. There is also a lower-bond temperature shown in Fig. 3D, below which grain-boundary diffusion itself can be suppressed. As a result, a *T*_1_ of 1300°C and a *T*_2_ of 1200°C proved optimal, allowing a very fine grain to achieve full densification of HEC.

Shrinkage by thermolysis can readily shorten the lattice constant of 3D structure HEC and enhance their resolution and mechanical properties. But the base of the structure is easily deformed during the heating process, as it turns into a distorted shape because of the adhesion between the bottom layer and the substrate (fig. S14, A and B), which is so-called anchoring effect (*38*). This has greatly hampered efforts to produce high-fidelity HEC nanolattice with uniform shrinkage. To overcome this challenge, we have quantitatively measured its pyrolysis shrinkage rates in different structural directions when critical dimension of polymer varies from 9 to 21 μm (Fig. 3E). For all structures, the shrinkage rates after sintering in the *x* and *y* directions are nearly equal, while the shrinkage rate in the *z* direction is bigger and increases with critical dimension. This indicates that the in-plane shrinkage (parallel to the substrate) of structures is relatively free and uniform, and out-plane shrinkage is not free and limited by anchored base, resulting in a larger value. When critical dimension decreases, the anchoring effect gradually weakens and disappears. Therefore, the in-plane shrinkage rate increases slightly, and out-plane shrinkage rate decreases to be nearly equal to in-plane shrinkage rate, which is about 52%. In view of this, we design the springs as sacrificial support structures to prevent direct contact between large structure and substrate (fig. S14C). As shown in Fig. 3F and fig. S14 (D and F), 3D nanolattices of HEC with varying critical dimensions all display uniform shrinkage and high fidelity. Based on the above optimized sintering process, we have fabricated fully dense and high-fidelity HEC nanolattices with feature size close to 150 nm (fig. S15).

### High-entropy–enhanced strength and ductility

HEC has gained substantial attention for their unique properties in mechanics, electricity, and thermodynamics (*39**–**42*). Dislocations are the internal line defects formed by the irregular arrangement of local atoms in crystal materials. Recently, it has been discovered that the complex interplay between dislocations and microstructures can improve the mechanical performance of ceramics (*43*, *44*). However, dislocations remain rare in oxide ceramics. In this work, we have shown that high-entropy strategies are a promising pathway for generating a high density of dislocations to promote enhanced strength and ductility in HEC.

**Fig. 4.**
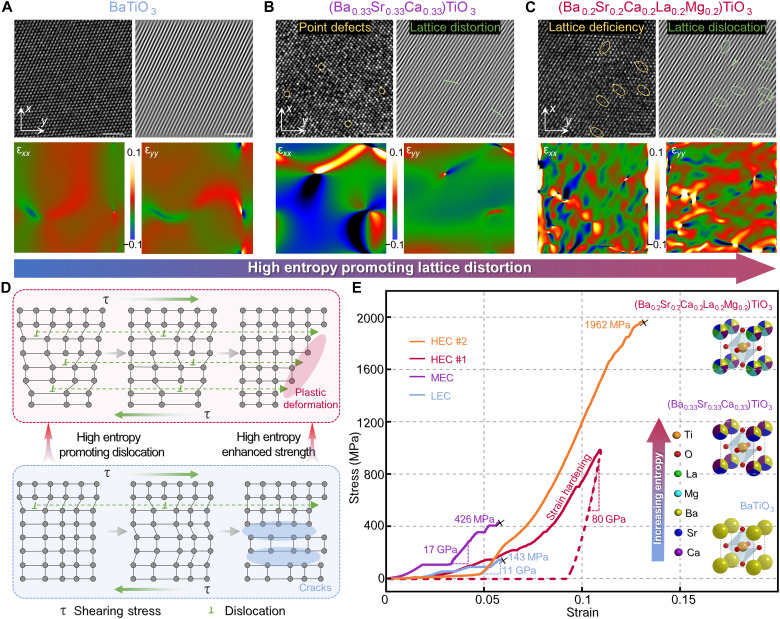
High-entropy enhancement mechanism related to lattice dislocations. (**A** to **C**) High-resolution TEM (HRTEM) images, the corresponding IFFT-filtered images, and the geometric phase analysis (GPA) mapping along the ε*_xx_* and ε*_yy_* directions of low-entropy BaTiO_3_, medium-entropy (Ba_0.33_Sr_0.33_Ca_0.33_)TiO_3_, and high-entropy (Ba_0.2_Sr_0.2_Ca_0.2_La_0.2_Mg_0.2_)TiO_3_, respectively. Colors from green to dark blue and red to bright yellow represent the compressive and tensile strains in the data bar, respectively. Scale bars, 2 nm and 2 nm^−1^, respectively. (**D**) Sketch map illustrating that drift and accumulation of edge dislocations under shear stress in the crystals that have different dislocation densities. (**E**) Comparison of stress-strain curves from micropillars with different entropies demonstrating high-entropy–enhanced strength and plasticity.

The high-resolution TEM (HRTEM) image of BaTiO_3_ clearly shows evident periodic lattices with few defects (Fig. 4A). The corresponding atomic inverse fast Fourier transform (IFFT)–filtered image with completely parallel stripes reveals the regular arrangement of atoms in a low entropy system. When Ba is replaced by half the molar amount of Sr in (Ba_0.5_Sr_0.5_)TiO_3_, clear lattice stripes remain visible, and the SAED pattern shows the standard tetragonal phase (fig. S16A). For the medium-entropy (Ba_0.33_Sr_0.33_Ca_0.33_)TiO_3_, the HRTEM image reveals the existence of some point defects in the periodic lattices (Fig. 4B). The IFFT-filtered image shows twisted stripes with a few distortions caused by increasing entropy. As exhibited in fig. S16C, a distinct grain boundary becomes apparent between the (100) and (110) planes of the (Ba_0.25_Sr_0.25_Ca_0.25_La_0.25_)TiO_3_ ceramic with a further increase in entropy. In the IFFT image, more structural defects, such as lattice distortions, can be observed (inserted map in fig. S16C). For the high-entropy (Ba_0.2_Sr_0.2_Ca_0.2_La_0.2_Mg_0.2_)TiO_3_ system, the HRTEM image and IFFT image both reveal prominent lattice point defects and discontinuous lattice fringes. These observations indicate the presence of entropy-driven dislocations with high density in the HEC (Fig. 4C). As shown in fig. S17, the dislocation density of HEC is around 10^9^ mm^−2^, which is much higher than the conventional oxide ceramics (~10^6^ mm^−2^). High-density dislocations in HEC can be observed even after high-temperature annealing (fig. S18), further suggesting that dislocations are stabilized by high entropy. Recent research also reveals that these dislocations are thermodynamically stabilized because the configuration-entropy gain can compensate for the large strain caused by the rigid ionic/covalent bonds in oxide ceramics (*32*, *45*). All these results demonstrate the vital role of configuration entropy of the complex ceramics on the stabilization of high-density dislocations. To further analyze the distorted lattice intuitively, the geometric phase analysis method is used on the basis of the HRTEM image (*46*). In the low-entropy system, the difference in atomic sizes is easily adapted through lattice volume changes. In contrast, the high-entropy system experiences cell deformation and significant shear strain due to different atomic deviations from ideal lattice sites and asymmetric distributions (*47*). As illustrated in the bottom of Fig. 4 (A to C), compared to low-entropy and medium-entropy ceramics, more lattice strains can be observed in the HEC along the ε*_xx_* and ε*_yy_* directions.

The high density of dislocations generated by high entropy is treated as the seed of plastic deformation to enhance the mechanical properties of HEC. Here, ceramic micropillars with different entropies are tested with in situ scanning electron microscopy (SEM) compression test. Stress-strain curves for low-entropy and medium-entropy pillars show the mechanism of “step-like” damage, which is related to brittle failure due to the low dislocation density. At the beginning of loading, stress increases synchronously with strain. However, the few dislocations are unable to provide timely and effective transfer channels for the microdeformation inside the ceramic (Fig. 4D). This results in excessive stress and local collapse, followed by stress release. Subsequently, the stress plateau period emerges until the internal voids caused by local collapse are filled with increasing strain (fig. S19 and movie S1). When loading continues, stress continues to increase with strain. Last, the ceramics fail with compressive stresses of 143 and 426 MPa, respectively. On the contrary, the high-entropy system generates the high-density lattice dislocations. Under stress, atomic bonds in dislocations break and then recombine to provide countless flexible and rapid transfer pathways for small deformations inside the lattice and avoid stress concentration (Fig. 4D) (*45*, *48*). As shown in Fig. 4E, the stress-strain curve of HEC #1 exhibits a large plastic deformation of 9% (Fig. 4E). To determine the real compressive strength of HEC micropillar at failure, another HEC sample (#2) is compressed to fail completely. For HEC #2 with complete failure, the accumulation of a large number of small deformations inside ceramics results in fracture strain of more than 0.1 and a compressive strength of 1962 MPa, which is more than 10 times that of low-entropy ceramics. This inherent combination of high strength and plasticity makes HEC very suitable to enhance mechanical metamaterials (fig. S20). It is worth noting that HEC exhibits the highest strength but almost lowest initial elastic modulus. This indicates that dislocation movement is very active at the beginning, resulting in significant strain strengthening mechanism and making HEC harder. Accordingly, a high elastic modulus of 80 GPa is observed from the unloading curve of HEC #1.

### High strength and energy absorption of HEC nanolattice

**Fig. 5.**
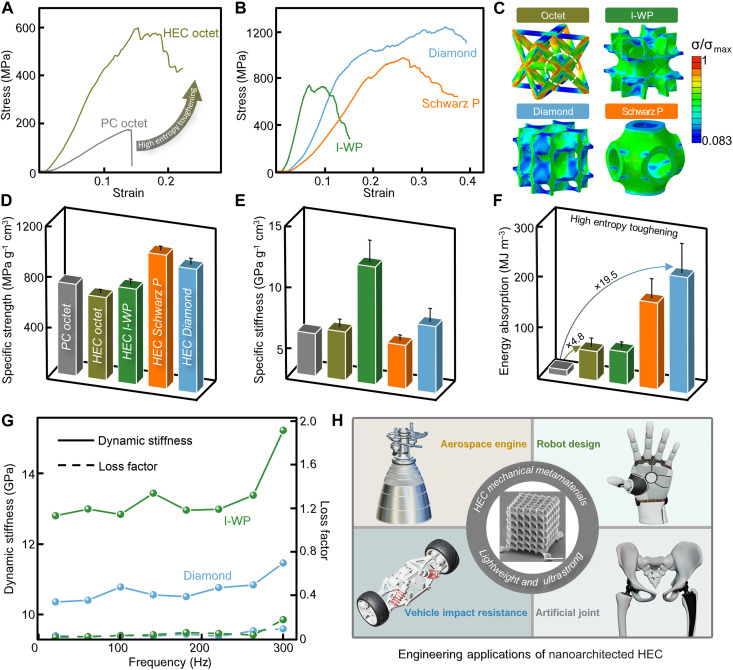
Mechanical properties of HEC nanolattices. (**A**) Stress-strain curves of HEC octet truss and PC octet truss. (**B**) Stress-strain curves of HEC shaped with Diamond, I-WP, and Schwarz P surface. (**C**) von Mises stress distribution, here normalized by the maximum stress value, of various unit cells with relative density of 20% at the 0.2% offset yield point. Specific strength (**D**), specific stiffness (**E**), and energy absorption (**F**) comparisons of PC octet, HEC octet, HEC I-WP, HEC Schwarz P surface, and HEC Diamond nanolattices. (**G**) Dynamic stiffness and loss factor vary as the functions of frequency in DMA of HEC. (**H**) Schematic of potential engineering applications of nanoarchitected HEC. Data are presented as means ± SD. Data in [(D) to (F)] are from *n* = 5 independent samples.

Recently, nanoscale 3D printing is making it possible for manufacturing lightweight materials with distinctive characteristics such as increased strength, tailored interactions with light or sound, and enhanced capacity for energy storage (*7*). For example, mechanical metamaterials, represented by PC nanolattices, have showed extremely high specific strength due to size effects and structural optimization related to power law scaling with relative density (figs. S21 and S22) (*49**–**51*). To demonstrate and compare the mechanical properties of as-fabricated HEC nanolattice with the most advanced PC nanolattices, a 3 × 3 × 3 HEC octet truss and a 3 × 3 × 3 PC octet truss are fabricated with the same structure size about 5 μm (movie S2). The relative density of all lattices is designed as 0.2 and corrected after sintering for calculation and simulation later (fig. S23). As shown in Fig. 5A, compared to PC octet with a brittle fracture, HEC octet exhibits a higher strength at a fracture strain over 0.2, indicating a high-entropy–enhanced toughness. We further fabricate various 3 × 3 × 3 HEC shell-based nanolattices (movie S3) and observe a highest strength of more than 1200 MPa with a failure strain close to 0.4 in Diamond nanolattice (Fig. 5B). Figure 5C displays the von Mises stress distribution of fabricated octet and shell-based cells from FE simulations at the 0.2% offset yield point. It is evident that the shell-based cells exhibit a more uniform stress distribution during deformation compared to the octet cell. Therefore, more parts simultaneously achieve the yield strength of the constituent materials in the shell-based nanolattice, which tends to improve the yield strength of the overall structure.

To further investigate mechanical properties of HEC nanolattices accurately, we performed a series of FE simulations of compressing samples, where the initial structure defects are applied by different percentages of buckling eigenmode of perfect models, and concrete damaged plasticity model is also used to simulate the high-entropy–enhanced plastic behaviors of HEC. Details for the FE simulations are provided in Materials and Methods. After introducing these initial defects, some parts of structures remain prebent or distorted before compression, which resembles structural defects in the experimental samples (fig. S24, A and B). As shown in fig. S25, the initial defects can reduce the compressive strength of nanolattices slightly, and the relative reduction in strength increases with greater initial defects. Nanolattices with octet cell are most susceptible to flaws, followed by Diamond, Schwarz P, and I-WP unit cell. All strain-stress curves with different initial defects are in good agreement with the experimental results, suggesting the repeatability of the mechanical properties under existence of some structure defects. FE results in fig. S24 also show that damage in the shell-based lattice is more distributed compared to octet where damage is seen to be more concentrated. Thus, compared to the catastrophic fracture of octet truss, shell lattices exhibit more stable crack growth when the strain increases, which matches the experimental results (movie S3). Detailed snapshots in fig. S24 (E and F) show that Schwarz P shell and I-WP lattices exhibit more complicated fracture characteristics and tortuous crack paths, with frequently observed crack deflection and crack branching. This reveals a higher driving force required for further crack propagation and a higher resistance to crack extension of shell lattice, which is also demonstrated in a previous work (*52*). These results from our current experimental and computational studies indicate that HEC nanolattices have simultaneous high strength and ductility, which is contributed by high-entropy effect as well as topology with nanoscale feature size.

Figure 5 (D to F) shows that specific strength and stiffness of HEC nanolattice are comparable to the PC octet with the same relative density, and the energy absorption improves by 4.8 to 19.5 times. When compared with advanced micro/nanolattices reported to date, as shown in fig. S26, HEC exhibits combination of various remarkable mechanical properties, such as specific strength of up to 1 GPa g^−1^ cm^3^, specific stiffness of up to 19 GPa g^−1^ cm^3^, and energy absorption of up to 230 MJ m^−3^ with larger fracture strain of 0.4. Last, DMA is used to exploring potential applications of HEC nanolattices (Fig. 5G). DMA results show that dynamic stiffness of HEC nanolattice increases with frequency and loss factor is nearly equal to zero, indicating that mechanical behavior of HEC structures is close to ideal elasticity without energy and stability loss under high frequency. These qualities make them ideal for lightweight and multifunctional structures working under harsh environments (high-frequency loading, high temperature, etc.) or complex miniaturized device (MEMs, etc.). In conclusion, it can be predicted that in the future, the lightweight HEC nanolattice with these remarkable static and dynamic mechanical properties is expected to gain great interest and be widely used as high-performance components in various emerging engineering applications like aerospace, robot design, vehicle impact resistance, and artificial joint (Fig. 5H).

## DISCUSSION

Nanoscale 3D printing is a powerful tool and has attracted considerable attention worldwide as it can create various properties that cannot be achieved in conventional materials (*53*). In the current study, we focused on broadening material compatibility of high-resolution 2PP 3D printing technology. To this end, a clever and innovative strategy is proposed for synthetizing various ceramic precursors (>15) that are compatible with high-resolution 2PP. Based on this versatile method, various functional ceramics with customizable constituents become possible (fig. S9). In addition, we successfully designed and fabricated 3D architected high-fidelity HEC nanolattice with feature size of 150 nm (Fig. 1). At the same time, we also provided a complete process on the basis of experiments and simulations for designing and processing multicomponent ceramic nanostructures (Fig. 2). Additionally, we adopted a two-step sintering approach specifically tailored for HEC densification and overcome anchoring effect during sintering, thus achieving stable and controllable 3D ceramic nanofabrication (Fig. 3). Although the topological structure and size effects used to enhance nanolattices have been extensively studied, efforts to seek harder and more robust constituent materials are still very limited. In this research, we demonstrated the generation of ultradense entropy-driven dislocation in HEC and experimentally indicated that it is a promising way to further strengthen ceramic nanolattices (Figs. 4 and 5). While the results are sound and promising, the investigation on high-entropy effect of ceramic nanolattices is still in its early stages. Considering the Hall-Petch relationship (*54*), grain size of HEC should be taken as a potential way to further strengthen ceramic nanolattices. Overall, this work provides profound insights into the exploration of the exceptional properties of new ceramics and broadens the application prospects of HEC in metamaterials, nanomechanical systems, and damage-resistant lightweight materials.

## MATERIALS AND METHODS

### Synthesis of various metal acrylates

All chemicals used are commercially available and used without further purification. Titanium ethoxide, niobium(V) ethoxide, strontium hydroxide (94%), calcium hydroxide, barium hydroxide monohydrate, lanthanum oxide, neodymium oxide, samarium oxide, europium oxide, magnesium hydroxide, lithium hydroxide, potassium hydroxide, sodium hydroxide, tin(II) ethoxide, basic lead carbonate, zinc hydroxide, indium hydroxide, acrylic acid (AAC), 2-methoxyethanol, and DETC are purchased from Shanghai McLean Biochemical Technology Co. Ltd. (Shanghai, China).

Except for the directly purchased metal acrylates, the rest are obtained by acid-base neutralization reaction between acrylic acid and corresponding metal oxides or hydroxides. The reaction mechanism involved is as follows

$$\begin{array}{c} \begin{array}{c} M{\left(\text{OH}\right)}_{m}/0.5\;M_{2}O_{m}+m\;\text{AAC}+n\;H_{2}O\;\overset{\text{ultrasonic}}{⟶}\;M-\text{AAC}\;\left(s\right)+H_{2}O \end{array} \end{array}$$

where m and n represent the amount of acrylic acid and water that generate a unit amount of the acrylic metal salt, respectively (fig. S7A). Subsequently, metal acrylates are dissolved in water by the coordination with acrylic acid to be fully transparent photosensitive ceramic precursors, which is described by the following expression

$$\begin{array}{c} M-\text{AAC}\;\left(s\right)+x\;\text{AAC}+y\;H_{2}O\;\overset{\text{ultrasonic}}{⟶}\;M-\text{AAC}\;\left(\text{aq}\right) \end{array}$$

where x and y refer to the amount of acrylic acid and water that dissolve a unit amount of the acrylic metal salt, respectively. More details about metal acrylates preparation are shown in table S5. All metal acylate precursors can be stably stored without precipitate for a long time (fig. S8A). Benefitting from material applicability, multicomponent ceramic resins can be prepared directly by mixing different precursors in proportion. The HEC resin consists of barium acrylate, strontium acrylate, calcium acrylate, lanthanum acrylate, magnesium acrylate, and titanium acrylate at a ratio of 1:1:1:1:1:5 and the photoinitiator DETC (2 wt %).

### Fabrication

#### 2PP of HEC resin

The femtosecond laser source used in our work is a Ti:sapphire laser oscillator (Chameleon Vision-S, Coherent Corp.) with a central wavelength of 800 nm, a pulse width of 75 fs, and a repetition rate of 80 MHz. The modulated beam propagates through a pair of 4f lenses before it is lastly focused by a high–numerical aperture (NA) oil-immersion objective (NA of 1.35, 60×, Olympus) into the HEC resin. All 3D structures are designed and generated using Blender modeling software, which are then sliced and converted into a dot matrix for printing. Specifically, all structures had 100-nm slicing distances and 150-nm hatched distances. In addition, structures with a critical dimension of more than 12 μm had 150-nm slicing distances and 200-nm hatched distances. To avoid the shadow effect of the previous aggregation structure on the incident light, we have used the “writing direction down” method (fig. S27). In the way of sapphire glass facing upward, the prepared sample is mounted on a nanopositioning stage (Physik Instrument, E727) with a nanometer resolution and a 200 μm–by–200 μm–by–200 μm moving range. All polymer nanolattices were printed at a laser power of 10 mW. For polymer nanolattices with a critical dimension of more than 12 μm, laser power was adjusted to 16 mW. The processed sample is immersed in 2-methoxyethanol for 30 min to remove the uncured resin.

#### Two-step sintering

The high-temperature tube furnaces used are GSL-1400X and GSL-1700X (Hefei Kejing Material Technology Co. Ltd.). Before two-step sintering, an additional debinding step is used to remove cross-linking polymer network gently and facilitate the shape retention of structures (*16*). The debinding process was undertaken in a tube furnace (Kejing GSL-1400X, China) in vacuum. During this process, the temperature was increased from room temperature to aim temperature with a ramping-up rate of 0.5°C min^−1^. The dwelling times were set as 180 and 120 min, respectively, when temperature reaching 350° and 550°C. After that, the samples were gradually cooled down to room temperature naturally. The sintering atmosphere is air, and only *T*_1_ and *T*_2_ are changed during the two-step sintering of all ceramic samples. Specifically, *T*_1_ and *T*_2_ are selected as 1300° and 1200°C for HEC samples, 1250° and 1100°C for medium entropy ceramics samples, and 1200° and 1050°C for low entropy ceramics samples, respectively. The temperature is raised to *T*_1_ at 10°C/min, then lowered to *T*_2_ at 20°C/min, and maintained for 3 to 20 hours. After the program is completed, it is cooled down with the furnace.

### Characterization

SEM: The SEM images are taken with a secondary electron scanning electron microscope (ZEISS EVO18, Germany) operating at an accelerating voltage of 10 keV. All samples are coated with gold using a gold evaporator for 80 s before tests. XRD: The XRD test is conducted using the Theta Rotating Anode X-ray Diffractometer (TTR-III) with Cu targets (Rigaku, Japan). The data are acquired within a 20° to 80° 2θ scan range using a LynxEye detector from the powdered samples of the ultraviolet-cured annealed preceramic resins. MDI JADE6 software [V6.2.9200; (*2*)] is used for baseline correction of data. EDS: EDS testing is conducted using a Schottky field emission scanning electron microscope (Gemini-SEM 360, Germany) equipped with the Bruker XFlash6160 under an accelerating voltage of 10 keV. XPS: High-resolution XPS with an energy step of 0.05 eV and full-spectrum XPS data with an energy step of 1 eV are both taken using a K-Alpha XPS instrument equipped with a monochromatic Al Kα x-ray source (Thermo Fisher Scientific, USA). For this analysis, 2D patterns (~100 μm by 100 μm) are printed and decomposed to ceramics on quartz glass slide substrates, and an Al Kα source of 200-μm spot size is used. XPS peak fitting data are generated by deconvoluting spectra using XPSPEAK41 software (V4.1). ATR-FTIR spectroscopy: FTIR spectrometer Nicolet 8700 (Thermo Scientific, USA) was used to collect spectra at the range of 400 to 4000 cm^−1^. Raman spectroscopy: Spectra were measured using a LabRAM SoLeil (Horiba, Japan) equipped with a 785-nm laser source. Thermogravimetry-differential scanning calorimetry (TG-DSC): Thermogravimetric analysis of HEC polymeric sample is performed using a Discovery SDT 650 (TA Instruments, USA) with temperature from 20° to 1400°C at a heating rate of 10°C/min in air. TEM: All ceramic sample cross sections with thickness of ~30 to 50 nm are fabricated using Ga+ FIB in the FEI Helios NanoLab 650 DualBeam SEM (Thermo Fisher Scientific, USA) equipped with an Omniprobe AutoProbe200. TEM images of all ceramic lattices are taken by a JEM-F200 microscope (JEOL, Japan) at 200 kV. All measurements and analyses are conducted in Gatan Microscopy Suite software (V3.22.1461.0). In situ SEM compression test: Compression test for samples with structural dimensions below 10 μm is performed on a NanoFlip nanoindenter (pressure range, 0 to 50 mN) with a flat punch diamond tip with diameter of 10 μm (KLA Corporation, USA) in a Gemini-SEM 360. For samples with structural dimensions of more than 10 μm, testing is conducted on a Hysitron PI 85E (pressure range, 0 to 10 mN) equipped with a conical shape 60° diamond tip with diameter of 50 μm (Bruker, Germany) in FEI Quanta 450 (Thermo Fisher Scientific, USA). All tests are performed at displacement rate of 5 nm/s for all cylindrical pillar samples and HEC samples with 3D microstructure. Load (millinewtons)–displacement (nanometers) curves are recorded. Applying the measured cross-sectional area of the top face of the structures, *A*, and the structure height, *H*, engineering stress and strain are determined. The compressive strength of the structures, σ, is defined as the maximum compressive stress before collapse. Their Young’s modulus, *E*, is determined as the maximum slope of the corresponding stress-strain curve. Nonlinear behavior at low strains is related to small misalignments between the indenter tip and the specimen, local surface roughness in the contact area, as well as structural defects of the specimen.

### First-principles calculation

In addition, first-principles calculation tools are adopted to verify the correctness of material design. Before theoretical calculation, all lattice models of ceramics are simulated using special quasirandom structures generated by the Alloy Theory Automation Toolbox (*55*). To ensure the accuracy of the calculation, we have selected a supercell scheme with 75 to 80 atoms (*56*). At the same time, considering the limitation of the indivisibility of atoms, that is, the number of atoms in the supercell model must be an integer, we build a monomer model containing five atoms and extend the unit cell to five supercells, which are 2 × 2 × 4 for BaTiO_3_, (Ba_0.5_Sr_0.5_)TiO_3_, and (Ba_0.25_Sr_0.25_Ca_0.25_La_0.25_)TiO_3_ and 1 × 3 × 5 for (Ba_0.33_Sr_0.33_Ca_0.33_)TiO_3_ and (Ba_0.2_Sr_0.2_Ca_0.2_La_0.2_Mg_0.2_)TiO_3_, respectively. All supercell models are visualized using VESTA software for display (fig. S4).

For preparation of theoretical calculation, all POSCAR files, containing the lattice geometry and the ionic positions, are obtained from bestsqs.out file when objective function decreases until it remains unchanged for a week. In this work, KPOINTS files for self consistent field (SCF) calculation, which specify the Bloch vectors (*k* points) used to sample the Brillouin zone, are generated using Monkhorst-Pack scheme with Kmesh-resolved value (in units of 2*Pi/angstrom) of 0.02. *K*-point meshes of different models are set to be 6 × 6 × 3 for BaTiO_3_, (Ba_0.5_Sr_0.5_)TiO_3_, and (Ba_0.25_Sr_0.25_Ca_0.25_La_0.25_)TiO_3_ and 13 × 4 × 3 for (Ba_0.33_Sr_0.33_Ca_0.33_)TiO_3_ and (Ba_0.2_Sr_0.2_Ca_0.2_La_0.2_Mg_0.2_)TiO_3_, respectively. The POTCAR files essentially contain the pseudopotential for each atomic species used in the calculation. Because total energies are calculated using density functional theory (DFT) as implemented in VASP using the Perdew-Burke-Ernzerhof (PBE) exchange-correlation functional (*57*), projector augmented wave–PBE potentials are applied with Ba-sv, Ca-sv, La-sv, Mg, O, Sr-sv, and Ti-sv (*58*, *59*). For all the calculations, a kinetic energy cutoff of 500 eV is used, and the global break condition for the electronic SC-loop (EDIFF) is set to be 10^−5^ eV. Geometry optimizations are performed using the conjugated gradient method, and the threshold (EDIFFG) is set at 10^−4^ eV/atom in energy (more details are shown in fig. S5).

### Calculation of dislocation density

To calculate the dislocation density, we count the number of dislocations in three independent TEM images for each composition. All the HRTEM images are taken at random positions of samples to reduce the randomness error. IFFT method is used to locate the specific position of dislocation. The dislocation density is then calculated to be the average number of dislocations per unit area (per square millimeter).

### Density of HEC

#### Calculating from the density of constituent oxides

The density of the high-entropy (Ba_0.2_Sr_0.2_Ca_0.2_La_0.2_Mg_0.2_)TiO_3_ ( $\rho_{\text{HEO}}$ ) is calculated as

$$\rho_{\text{HEO}}=\sum_{i=1}^{N}\left(w_{i}\rho_{i}\right)$$

where $w_{i}$ and $\rho_{i}$ are wt % and density of monometallic oxide, respectively. The density of BaO, CaO, SrO, La_2_O_3_, MgO, and TiO_2_ are ~5.72, ~3.35, ~4.70, ~6.51, ~3.58, and 4.29 g/cm^3^, respectively. The wt % is defined as the monometallic oxide’s mass proportion of HEC, which is composited with monometallic oxides in terms of material composition ratio: 1 mol of TiO_2_ and 0.2 mol of BaO, CaO, SrO, La_2_O_3_, and MgO. The density of high-entropy (Ba_0.2_Sr_0.2_Ca_0.2_La_0.2_Mg_0.2_)TiO_3_ is calculated as 4.88 g/cm^3^.

#### Calculating from the XRD refinement lattice constants

Through XRD refinement, the lattice constants of HEC are figured out, *a* = *b* = 3.9265 and *c* = 2.8806 (Fig. 1C). In a HEC lattice, there are three oxygen atoms and one titanium atom, and the remaining atomic position is equally likely to be occupied by barium, strontium, calcium, lanthanum, and magnesium atom. Based on the above, the density of the high-entropy (Ba_0.2_Sr_0.2_Ca_0.2_La_0.2_Mg_0.2_)TiO_3_ ( $\rho_{\text{HEO}}$ ) is calculated as

$$\begin{array}{c} \rho_{\text{HEO}}=\frac{3M_{O}+M_{\text{Ti}}+\left(M_{\text{Ba}}+M_{\text{Sr}}+M_{\text{Ca}}+M_{\text{La}}+M_{\text{Mg}}\right)\;/\;5}{N_{A}\times \mathrm{abc}} \end{array}$$

where $M_{O}$ , $M_{\text{Ti}}$ , $M_{\text{Ba}}$ , $M_{\text{Sr}}$ , $M_{\text{Ca}}$ , $M_{\text{La}}$ , and $M_{\text{Mg}}$ and $N_{A}$ are the relative atomic mass of the subscript atoms and the Avogadro constant, respectively. The density of high-entropy (Ba_0.2_Sr_0.2_Ca_0.2_La_0.2_Mg_0.2_)TiO_3_ is calculated as 5.03 g/cm^3^. Last, we determine the density of HEC as (4.88 + 5.03)/2 = 4.95 g/cm^3^.

### FE simulations

All geometrical models are reconstructed in SolidWorks 2018 (Dassault Systèmes SE) with SEM-measured wall and beam dimensions of the sintered HEC structures (fig. S23). Then, the computer aided design (CAD) model is exported as STEP AP203 file and then imported as a part in Abaqus. FE simulation is performed in ABAQUS (Dassault Systèmes SE) to model the compression process of the HEC nanolattices domes.

To study the effect of HEC on cracks propagation, we use concrete damaged plasticity model that is also suitable to analyze ceramic materials (*60*). This model considers both tensile and compressive cracking and allows separate tensile and compressive behaviors. Damage is characterized by a scalar degradation variable *d* = (1 − *d*_t_) (1 − *d*_c_), where *d*_t_ and *d*_c_ are compressive and tensile degradation variables and are functions of plastic strains. As the material degrades, the elastic modulus of the material decreases. Two stress invariants are used in flow potential and yield function in this model. A nonassociated flow rule is used where the plastic potential function is the Drucker-Prager hyperbolic function. Dilation angle and flow potential eccentricity are two input parameters of the plastic potential function, which are assumed as the values of 31° and 0.1, respectively. The shape factor and ratio of biaxial to uniaxial compressive strengths are two input parameters that describe yield function, and we set values of 0.66 and 1.16 to them, respectively. We set values listed in table S6 for the yield and degradation behavior in both compression and tension of the material. In linear elastic region, material is modeled with Young’s modulus of 300 GPa, a yield stress of 2000 MPa, and a Poisson’s ratio of ν = 0.2.

Before compression, we introduced initial structural defects to the simulated nanolattices by imposing the corresponding buckling eigenmodes of nanolattices. These eigenmodes of nanolattices are obtained from the FE linear buckling analysis. The maximum defects of the structures are set as 0, 10, 20, and 40% of the first eigenmode (fig. S24, A to D).

The contact algorithm is set as “general contact,” which means that every face can contact each other based on a real situation. The tangential friction coefficient is set as 0.1 and the expected behavior as hard contact considering the real situation. For pressure plates and specimens, the constraint type is set as “tie” with discretization method of surface to surface. The bottom boundary is set as a fully rigid boundary condition in simulation according to the real condition, and the top is imposed by displacement loading. The displacement applied in the model is 30% of the overall size of the structure. Here, the explicit dynamic analysis is used to simulate the quasistatic compression process. The loading speed is carefully chosen to ensure that the ratio of kinetic energy to internal energy remains below 5%. Based on mesh sensitivity analysis, the models were meshed with ~20,000 elements per unit cell for octet lattices and ~100,000 elements per unit cell for shell-based lattices via four-noded linear tetrahedron elements (C3D4). The force-displacement curve is obtained from the output of the object database format (ODB) history output.

## Supplementary Materials

### The PDF file includes:

Supplementary Text Figs. S1 to S27 Tables S1 to S6 Legends for movies S1 to S3 References
