# Supplementary material for: Lightweight and ultrastrong 3D nanoarchitected high-entropy ceramic metamaterials
Source: Sci Adv. 2025 Oct 17;11(42):eadw6632. doi: 10.1126/sciadv.adw6632 (PMC12533655; doi:10.1126/sciadv.adw6632)
Supplement: Supplementary file 1 — Supplementary Text Figs. S1 to S27 Tables S1 to S6 Legends for movies S1 to S3 References [file sciadv.adw6632_sm.pdf]

Science Advances
AAAS

Supplementary Materials for

Lightweight and ultrastrong 3D nanoarchitected high-entropy ceramic metamaterials

Modong Jiang *et al.*

Corresponding author: Jiawen Li, jwl@ustc.edu.cn

Sci. Adv. **11**, eadw6632 (2025)
DOI: 10.1126/sciadv.adw6632

The PDF file includes:

Supplementary Text
Figs. S1 to S27
Tables S1 to S6
Legends for movies S1 to S3
References

Other Supplementary Material for this manuscript includes the following:

Movies S1 to S3

Supplementary Text

Calculation of Mixing entropy

Mixing entropy (ΔS_{mix}) is originating from disorder in crystals and enhances when more metal elements are added to the $BaTiO_3$-based system. The constructed HEC with equal molar fractions of each element in A site will maximize the ΔS_{mix} values to be 1.69R, over 1.5R, eligible to be defined as high entropy. The configurational entropy of material is calculated by the following equation(*28*):

$$\Delta S_{mix} =- R[\left(\sum_{i=1}^{N} x_i \ln x_i\right)_{cation-site} + \left(\sum_{j=1}^{M} x_j \ln x_j\right)_{anion-site}]$$

where *R, N (M)*, and x_i *(*x_j*)* refer to the gas constant, atomic species, and contents at the cation (anion) sites, respectively.

$BaTiO_3$ is calculated as following:

$$\Delta S_{mix}(BaTiO_3) =- R(\ln 1) = 0$$

$(Ba_{0.5}Sr_{0.5})TiO_3$ is calculated as following:

$$\Delta S_{mix}((Ba_{0.5}Sr_{0.5})TiO_3) =- R(0.5 \ln 0.5 + 0.5 \ln 0.5) = 0.69R$$

$(Ba_{0.33}Sr_{0.33}Ca_{0.33})TiO_3$ is calculated as following:

$$\Delta S_{mix}((Ba_{0.33}Sr_{0.33}Ca_{0.33})TiO_3) =- R(0.33 \ln 0.33 + 0.33 \ln 0.33 + 0.33 \ln 0.33) = 1.1R$$

$(Ba_{0.25}Sr_{0.25}Ca_{0.25}La_{0.25})TiO_3$ is calculated as following:

$$\Delta S_{mix}((Ba_{0.25}Sr_{0.25}Ca_{0.25}La_{0.25})TiO_3)$$
$$=- R(0.25 \ln 0.25 + 0.25 \ln 0.25 + 0.25 \ln 0.25 + 0.25 \ln 0.25) = 1.39R$$

$(Ba_{0.2}Sr_{0.2}Ca_{0.2}La_{0.2}Mg_{0.2})TiO_3$ is calculated as following:

$$\Delta S_{mix}((Ba_{0.2}Sr_{0.2}Ca_{0.2}La_{0.2}Mg_{0.2})TiO_3)$$
$$=- R(0.2 \ln 0.2 + 0.2 \ln 0.2 + 0.2 \ln 0.2 + 0.2 \ln 0.2 + 0.2 \ln 0.2) = 1.61R$$

All calculation results for ΔS_{mix} are summarized in table S1.

Calculation of Gibbs free energy

Generally, the phase stability of the multicomponent system can be evaluated based on the Gibbs free energy formula. The Gibbs free energy (ΔG_{mix}) of high-entropy ceramic can be calculated according to the following formula(*29*):

$$\Delta G_{mix} = \Delta H_{mix} - T\Delta S_{mix}$$

where ΔH_{mix} is the mixing enthalpy, ΔS_{mix} is the mixing entropy, and T is the thermodynamic temperature. Notably, a negative ΔG_{mix} suggests that a single-phase state is prone to form, whereas a positive value indicates thermodynamic phase separation. All calculation results for ΔG_{mix} are summarized in table S1 and shown in fig. S5.

In addition, the enthalpy of mixing (ΔH_{mix}) is the difference between the total energy of the polymetallic ceramics and the total energy of each monometallic ceramics given by density functional theory (DFT). The entropy (ΔS_{mix}) can be calculated from the configurational entropy. It is calculated by the following formula(*61*):

$$\Delta H_{mix} = E_{multi-metal} - \sum_{i=1}^{N} (x_i E_{mono-metal})$$

$$\Delta S_{mix} =- \frac{k_B}{2} \sum_{i=1}^{N} (x_i \ln x_i)$$

where $E_{multi-metal}$ is the total energy of polymetallic ceramics, $E_{mono-metal}$ is the energy of various monometallic ceramics. x_i is referred to the atomic percent of one transition metal in the

overall transition metals. N is the number of transition metals in the high-entropy ceramics. k_B is Boltzmann constant.

Calculation of Goldschmidt tolerance factor

For HEC with targeted perovskite phase (ABX_3), the Goldschmidt tolerance factor, $t_{effective}$ is a more appropriate criterion for determining whether a multi-component system can form a stable crystal phase. It measures the deviation of lattice composited of ions with different radius from the standard cubic phase of which value is $t_{effective} = 1$. $t_{effective}$ is defined as follows(*30*):

$$t_{effective} = \frac{r_A + r_X}{\sqrt{2}(r_B + r_X)}$$

where r_A and r_B are the ionic radius of the A and B site cations respectively, and r_X is the ionic radius of the anion. Since there is more than one element at the A or B positions, the "estimated effective ion radius" ($r_{effective}$) needs to be calculated by weighting the ion ratio:

$$r_{effective} = \sum_{i}^{n} x_i r_i$$

where n is the number of atomic species, x_i is the atomic contents, r_i is the ionic radius. As shown in table S1 and fig. S6B, all the perovskite ceramics and HEC may exist stably due to each $t_{effective}$ ranging from 0.8 to 1.

Calculation of lattice size difference

To introduce various metal atomic with different size into A site, the targeted HEC lattice must undergo controllable contraction or elongation. Lattice size difference, δ, has been commonly accepted to represent the effects of the atomic-size difference on the structural stability. δ values change with multiple elements (Sr, Ca, La and Mg) introduced into the Ba sites (table S1). δ (%) is calculated by the following equation(*31*):

$$\delta = \sqrt{\sum_{i=1}^{n} c_i (1 - \frac{r_i}{\sum_{i=1}^{n} c_i r_i})^2}$$

where n is the number of atomic species, c_i is the atomic contents, r_i is the atomic radius. All calculation results for δ are summarized in table S1.

Geometry optimizations in VASP

During the calculations, we change the ISIF to determine if the stress tensor is calculated and which ionic degrees of freedom are varied. Specifically, we first fix the cell volume and make ion positions and cell shape relax (ISIF=7). Then we let cell volume, ion positions and cell shape relax together (ISIF=3) to make lattice to tend towards final stability. For each ISIF value, the calculation terminates when the lattice constant error (ε) of the last two geometry optimizations is less than 10^{-3}, defined as:

$$\varepsilon = \left| \frac{d_n - d_{n-1}}{d_{n-1}} \right|$$

where d_n and d_{n-1} are lattice constant of No.n and No.$n-1$ geometry optimizations. The changes in lattice parameters during the geometry optimizations are shown in fig. S5.

Characteristic length of ceramic materials

Existing experimental studies have demonstrated that brittle materials become insensitive to flaws when their dimensions are reduced below a critical size, driving their strength toward the theoretical limit(*6, 23*). Inspired by bone-/nacre-like biological materials with high strength and fracture toughness due to their nanoscale mineral platelets, characteristic length is considered as another barrier that hinders HEC nanolattices from being ultra-strong nanoceramic materials. The fracture strength of a mineral platelet is calculated according to the Griffith theory as(*62*):

$$\sigma_f = \sqrt{4\gamma E/(1-\nu^2)h}$$

where h is the width of the platelet, γ is the fracture surface energy, E is the Young's modulus, and ν is the Poisson's ratio. Based on this equation, the strength increases with decreasing h. This is also known as the "smaller and stronger" effect. In large-sized materials, defects that follow statistical distribution have a high probability of being discovered, leading to material failure under lower stress. Materials with micro and nano sizes have a narrow distribution of defect sizes, greatly reducing the probability of discovering defects. When the size is further reduced to the critical size (h_{cr}), it becomes insensitive to defects and can theoretically achieve near atomic bond strength. For most brittle materials like ceramics, the critical length h_{cr} for flaw insensitivity is on the order of tens to hundreds of nanometers.

Discussion of scaling of modulus and strength with relative density of different geometries

For porous structure, the cost of ultra-lightweight is to significantly reduce its stiffness and strength through power-law scaling: $\sigma_y \sim (\rho/\rho_s)^m$, $E \sim (\rho/\rho_s)^n$, where σ_y is the yield strength, E is the Young's modulus, ρ is density of the porous structure, ρ_s is the density of a completely dense solid and parameter m, n are constants dependent on the architecture(*5, 50*). In addition, ρ/ρ_s is also defined as relative density ($\bar{\rho}$). For an ideal stretching-dominated structure, the scaling indices m and n are equal to 1. For bending-dominated structures, m=2 and n=1.5(*63*). In general, stretching-dominated topologies are more mechanically efficient(*1, 50*).

"Writing direction down" strategy

The enlarged illustration of fig. S27A shows a type of TPP processing strategy called "writing direction down" which is compared to the standard approach. For the standard approach, laser beam is initially focused at the lower glass/resin interface, and successively moved layer-by-layer at increasing z-coordinates for polymerizing the whole structure. Power compensation is also required for assuring a constant exposure dose at increasingly higher penetration depths within the liquid and through just polymerized layers. "Writing direction down" strategy is developed to solve these problems: a preceramic polymer solution drop is placed between an ordinary glass substrate and a sapphire glass slide, separated by a thin polydimethylsiloxane (PDMS) membrane serving as gasket. The laser beam is initially focused at the upper glass/resin interface, and fabrication proceeds in a layer-by-layer fashion for decreasing z-coordinates, eliminating shadowing effects from previously polymerized layers.

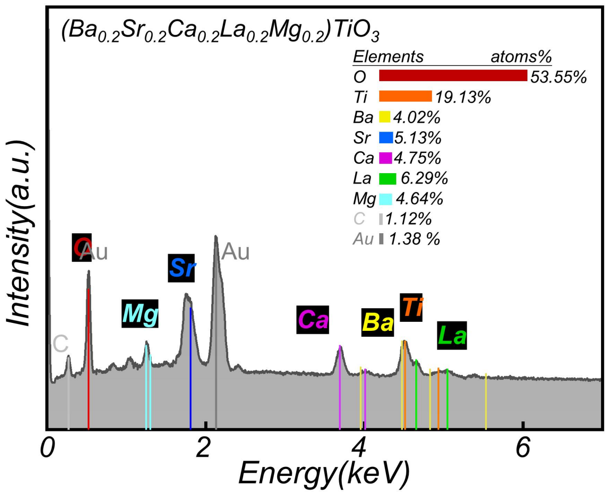

Fig. S1. EDS spectrum map of ($Ba_{0.25}Sr_{0.25}Ca_{0.25}La_{0.25}$)$TiO_3$. The EDS spectrogram of high-entropy oxide ceramic ($Ba_{0.2}Sr_{0.2}Ca_{0.2}La_{0.2}Mg_{0.2}$)$TiO_3$ demonstrates the content of A-site elements is close to equal proportion. The detected Au (~ 5 nm thickness) is introduced to enhance the conductivity of the EDS sample. And a trace of C is observed in the sample due to possible pollution source include combustion residue and SEM chamber deposits(*64*). The result proves that proposed method can basically prepare HEC without impurities, which may weaken the mechanical properties of the HEC nanolattice. In some case, however, different existence forms of carbon can enhance the mechanical performance of metamaterials. For example, carbon nanomaterials such as graphene, carbon nanotubes and nano diamond, are known for their high intrinsic strengths and Young's modulus(*65-68*). Especially, a recent reported work demonstrated a high-strength, and high-ductility hybrid-carbon lattice via partial carbonization, breaking the preconceived notion of fully pyrolyzed lattice materials(*69*). The ductility enhancement is thanks to the collaborative contribution from its constituents of different carbon. Overall, it is potential to create metamaterials with tailored mechanical properties by adjusting contents and forms of carbon.

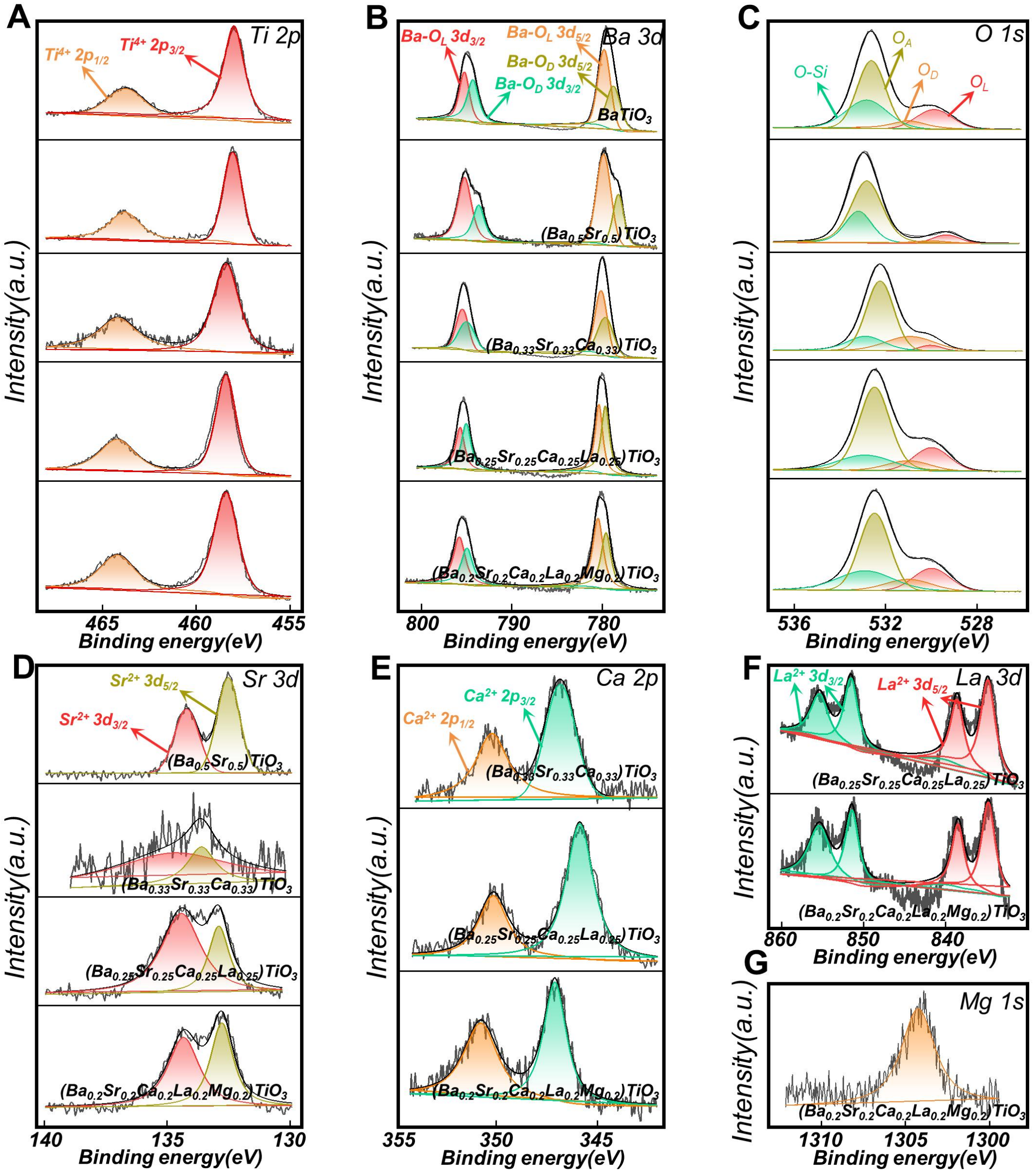

Fig. S2. X-ray photoelectron spectroscopy of Ti, Ba, O, Sr, Ca, La and Mg. (**A**) The spectrogram of Ti 2p indicates the presence of Ti-O (458.41 and 464.16 eV for Ti 2p3/2 and Ti 2p1/2, respectively). (**B**) The spectrogram of Ba 3d illustrates that the Ba 3d5/2 and Ba 3d3/2 peaks are matched by two different peaks. The main peaks correspond to Ba atoms in the configuration of ABO_3 perovskite, whereas the slight peaks are associated with Ba atoms in the non-perovskite structure(*70, 71*). (**C**) The O 1s spectra is deconvoluted into four species, namely, silicon-oxygen bond (Si-O) at 532.9 eV, absorbed surface water (O_A) at 532.5 eV, oxygen vacancy defects (O_D) at 531.0 eV and lattice oxygen (O_L) at 529.9 eV(*72*). (**D**) High-resolution Sr 3d XPS characteristic spectrum indicates the existence of Sr-O (132.83 and 134.38 eV for Sr 3d5/2 and Sr 3d3/2, respectively). (**E**) High-resolution Ca 2p XPS characteristic spectrum detects the presence of Ca-O (347.09 and 350.82 eV for Ca 2p3/2 and Ca 2p1/2, respectively). (**F**) In high-resolution La 3d XPS characteristic spectrum, two spin orbital peaks, La 3d5/2 at 834.56 eV and La 3d3/2 at 851.27 eV, can be seen in the La 3d spectra. Due to the complicated

architectures of the two constituents, the peaks centered at 855.26 and 838.28 eV are assigned as 3d3/2 and 3d5/2 satellite peaks, respectively(*73, 74*). (**G**) In high-resolution Mg 1s XPS characteristic spectrum of $(Ba_{0.2}Sr_{0.2}Ca_{0.2}La_{0.2}Mg_{0.2})TiO_3$, Mg-O (1304.26 eV for Mg 1s) is clearly observed.

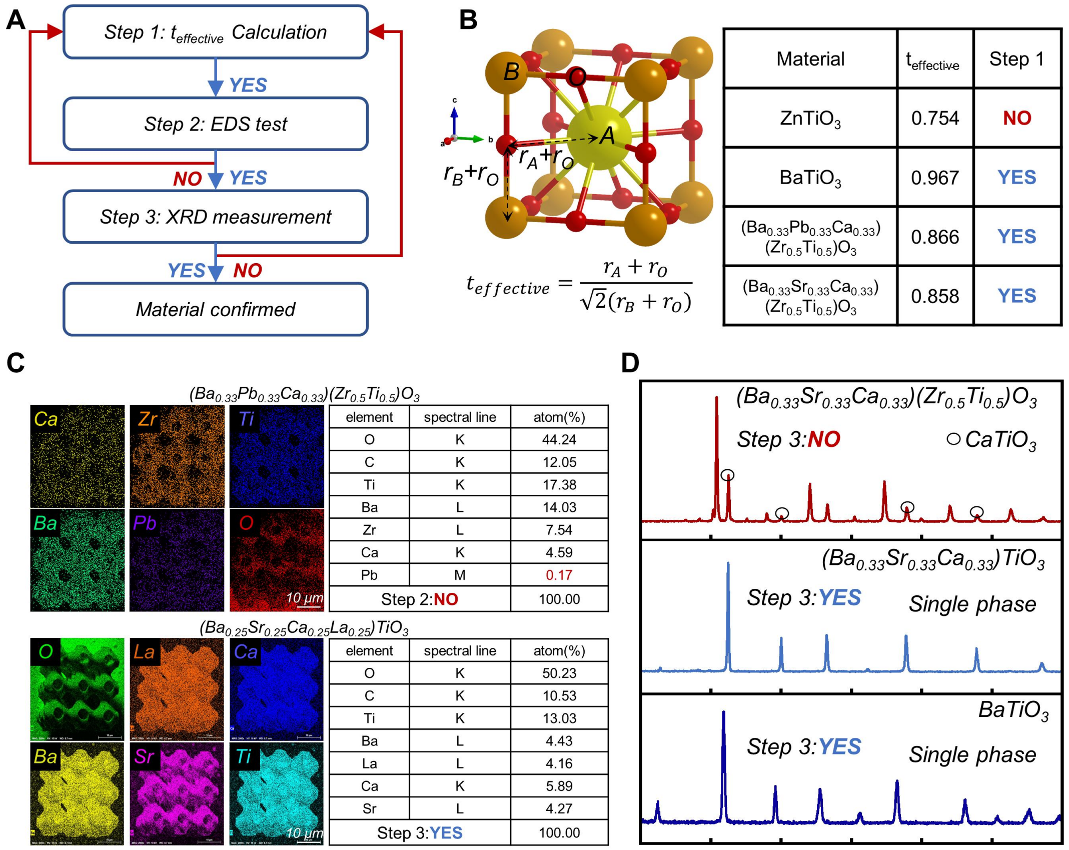

Fig. S3. Component design and optimization of perovskite-type HEC. Process diagram for quickly confirming HEC components with a single perovskite crystal phase. (**A**) Loop iteration design block diagram used to determine the final HEC formula. (**B**) Schmidt tolerance factor is one of the effective criteria for determining the stable formation of perovskite structures(*75*). Generally, the value between 0.8 and 1 indicates thermodynamic stability of the perovskite crystal phase. Due to the small radius of zinc ions, the $ZnTiO_3$ lattice is far away from the standard cubic phase, which means that perovskite crystal phase cannot be formed. (**C**) According to the definition of configuration entropy, proportional components can maximize the mixing entropy of the system. With the assistance of Energy Dispersive Spectrometer (EDS), we can intuitively determine whether the components in the material system have equal content. The volatility of lead makes it difficult for lead based HEC to meet the requirements of equal proportion components. Scale bar: 10μm. (**D**) Last but not least, it is important to use x-ray diffraction (XRD) to confirm the crystal phase of HEC candidates and compare it with the standard card. It is not difficult to observe that the introduction of zirconium ions reduces the solubility of calcium ions in the perovskite crystal phase system, resulting in the formation of impurity, calcium titanate.

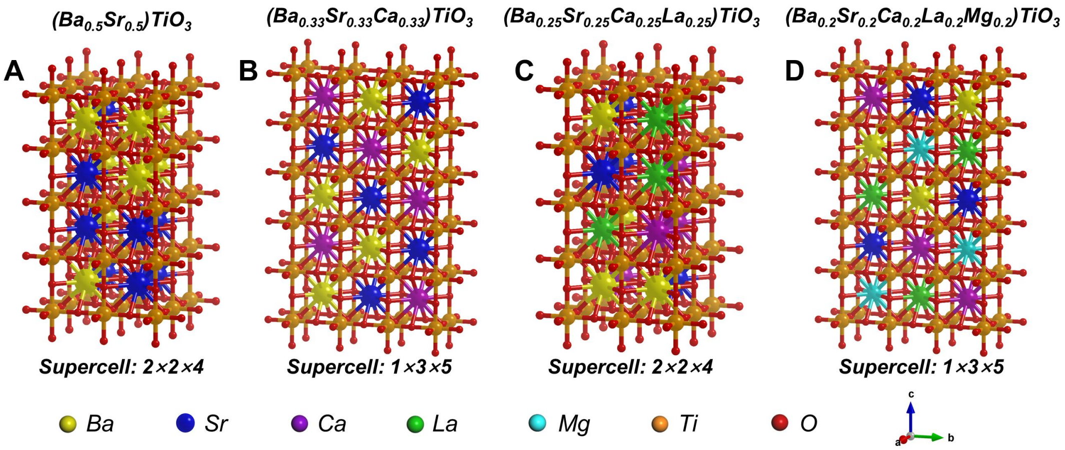

Fig. S4. Modeling of disordered solid solutions. The supercell models of $(Ba_{0.5}Sr_{0.5})TiO_3$ 2×2×4 (**A**), $(Ba_{0.33}Sr_{0.33}Ca_{0.33})TiO_3$ (**B**) 1×3×5, $(Ba_{0.25}Sr_{0.25}Ca_{0.25}La_{0.25})TiO_3$ 2×2×4 (**C**) and high entropy Oxide Ceramic $(Ba_{0.2}Sr_{0.2}Ca_{0.2}La_{0.2}Mg_{0.2})TiO_3$ 1×3×5 (**D**) show the solid-solution state of Ba, Sr, Ca, La, Mg in the A site.

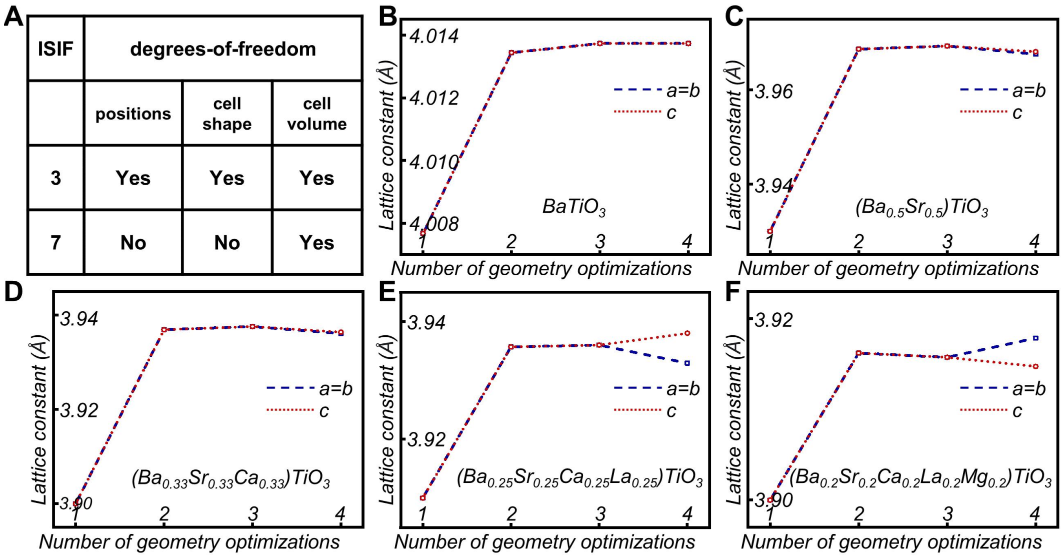

Fig. S5. Geometry optimizations of ceramics with different entropies. (**A**) ISIF determines which degrees of freedom (ionic positions, cell volume, and cell shape) of the structure are allowed to change in calculation. For ISIF=3, ionic positions, cell volume and cell shape are allowed to vary together. For ISIF=3, cell volume is allowed to vary while ionic positions and cell shape are fixed. (**B** to **F**) Processes for geometry optimization of $BaTiO_3$, $(Ba_{0.5}Sr_{0.5})TiO_3$, $(Ba_{0.33}Sr_{0.33}Ca_{0.33})TiO_3$, $(Ba_{0.25}Sr_{0.25}Ca_{0.25}La_{0.25})TiO_3$ and $(Ba_{0.2}Sr_{0.2}Ca_{0.2}La_{0.2}Mg_{0.2})TiO_3$, respectively. For all systems, the optimization with ISIF of 7 is conducted three times and the last optimization with ISIF of 3 obtains the final lattice parameters. The final lattices is determined when the constants error of the last two geometric optimizations is within 10^{-3}. The calculation results show that all ceramic crystal phases are tetragonal ($a = b \neq c$).

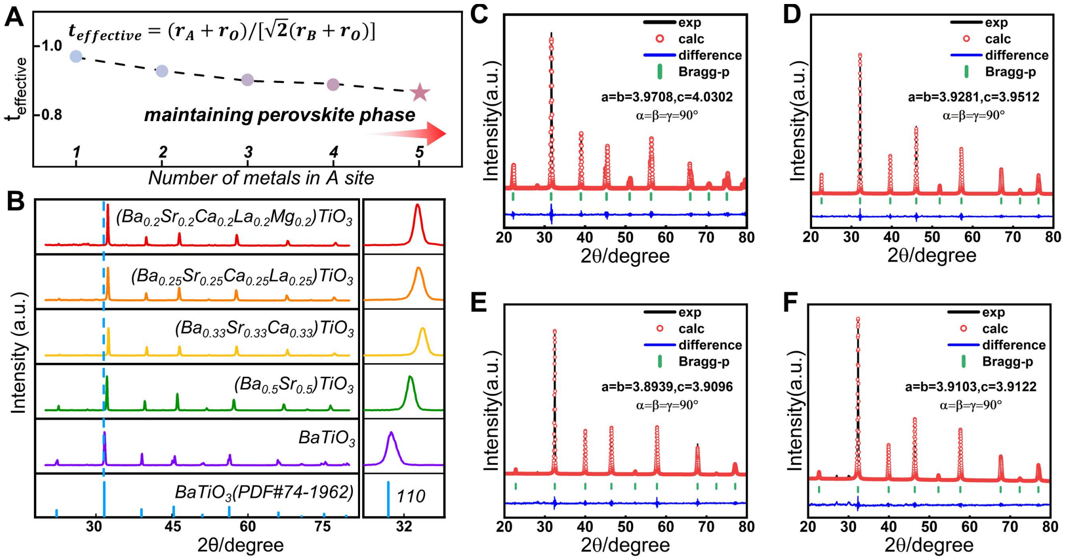

Fig. S6. Theoretical design and experimental confirmation of $BaTiO_3$-based HEC. (**A**) Goldschmidt tolerance factor as function of the number of metals indicates that perovskite-type HEC is theoretically stable and capable of maintaining single-phase with increasing number of metals in A site of titanate perovskites. (**B**) XRD patterns of the titanate perovskites + Ba, Sr, Ca, La, and Mg species revealed that all the $BaTiO_3$-based perovskites we synthesized have a P4mm structure. At the same time, the introduction of numerous metal elements, which have smaller cation radii than that of Ba^{2+} (table S4), gives rise to lattice contraction (associated with movement of X-ray diffraction peaks to higher angles). Rietveld refinement patterns of $BaTiO_3$ ceramic (**C**), $(Ba_{0.5}Sr_{0.5})TiO_3$ ceramic (**D**), $(Ba_{0.33}Sr_{0.33}Ca_{0.33})TiO_3$ ceramic (**E**) and $(Ba_{0.25}Sr_{0.25}Ca_{0.25}La_{0.25})TiO_3$ ceramic (**F**) indicate the lattice constant varies slightly with different atoms occupying the A site. Generally speaking, the introduction of larger ion radii leads to an increase in lattice constant, while smaller ion radii lead to the opposite result.

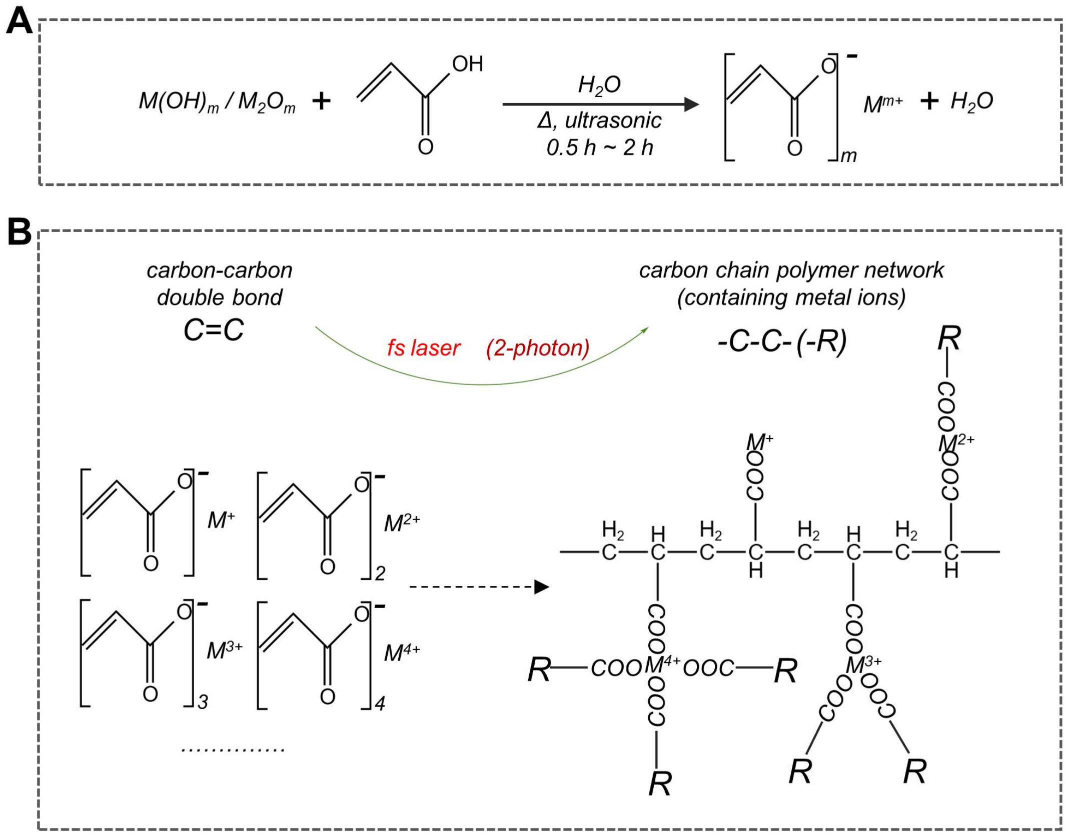

Fig. S7. Synthesis and polymerization mechanism of metal acrylates. (**A**) The reaction equation for synthesizing metal acrylates, utilizing the acid-base neutralization reaction between acrylic acid and metal hydroxides or oxides. (**B**) Schematic illustration showing 3D nano-printing of metal acrylates. Under two-photon excitation, the carbon-carbon double bonds in the acrylic acid salt open and connect with each other to form carbon chain polymer network (containing metal ions). The degree of network cross-linking is promoted by high valence metal ions (>2+) which are linked to multiple acrylic acid groups. As a result, there is no need to add excess crosslinking agents (such as PETA, PEGDA).

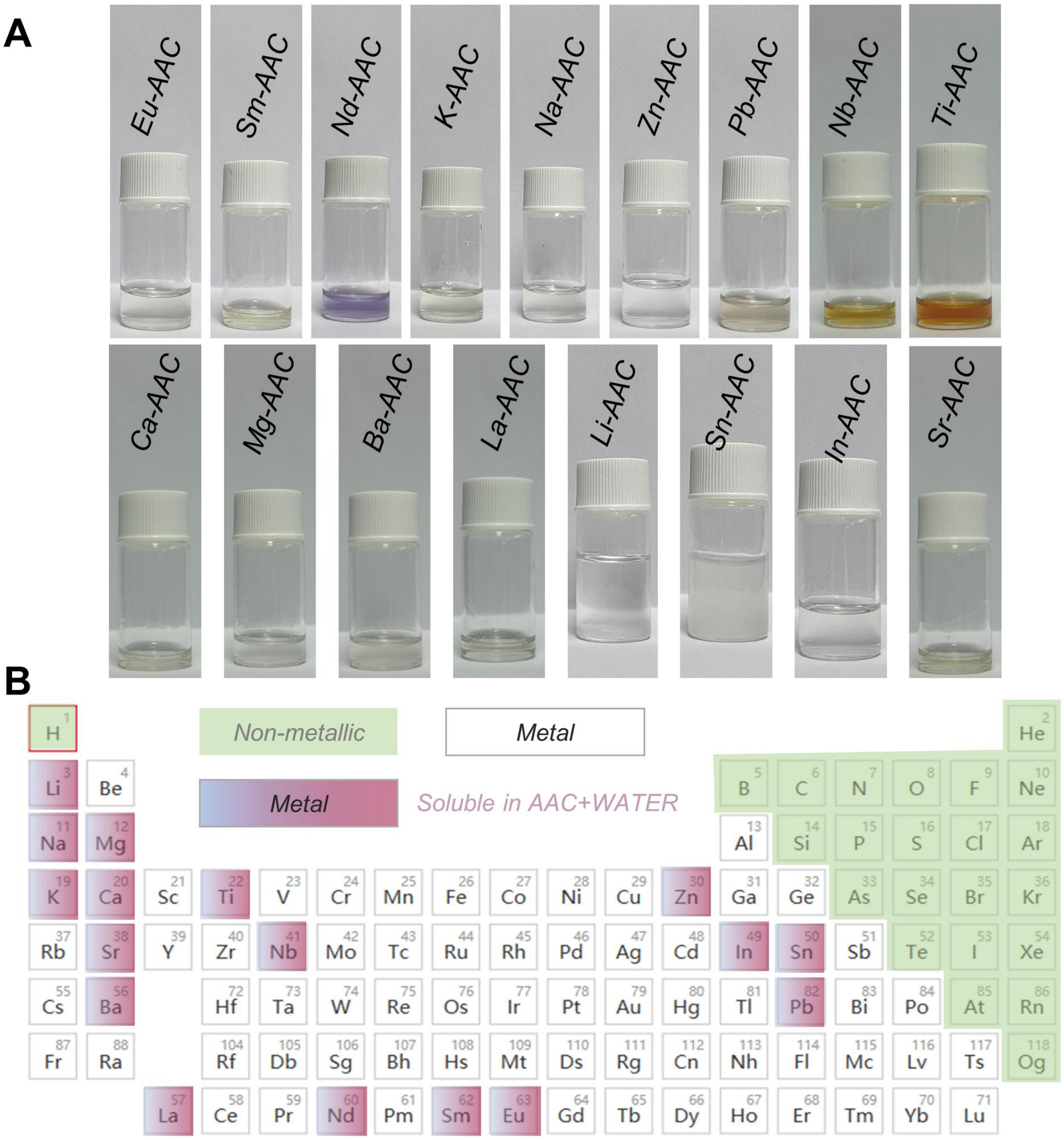

Fig. S8. Available metal acrylates material library. (**A**) Photographs of completely transparent solutions of various metal acrylates dissolved in AAC + water. (**B**) We propose a universal strategy for preparing thoroughly transparent metal acrylate solutions, which covered various metal elements of different ethnic groups with more than 15. There is every reason to believe that this method can prepare more metal acrylates solutions for manufacturing various types of oxide or ceramic micro functional devices.

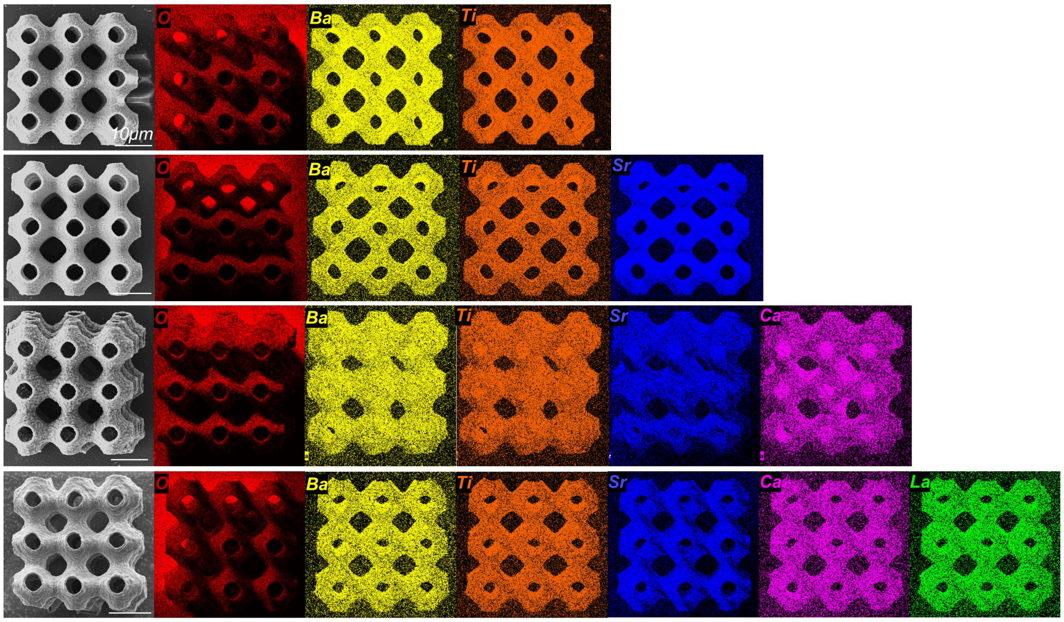

Fig. S9. Other oxide ceramics nanolattices fabricated in this work. The EDS mapping images of $BaTiO_3$ ceramic, $(Ba_{0.5}Sr_{0.5})TiO_3$ ceramic, $(Ba_{0.33}Sr_{0.33}Ca_{0.33})TiO_3$ ceramic and $(Ba_{0.25}Sr_{0.25}Ca_{0.25}La_{0.25})TiO_3$ proved the uniform distribution of various metal elements in ceramic microstructures. Scale bars: 10 μm.

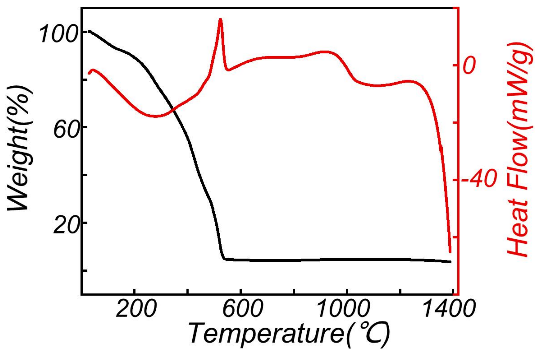

Fig. S10. TG-DSC thermal analysis curve of pre-HEC polymer. From rome temperature to approximate 550 °C, volatile species leave the material while the polymer scaffold decomposes to various metal oxides. When temperature continus to rise to 1100 °C (or higher), the metal oxides transform into HEC with a single ceramic phase (solid solution system).

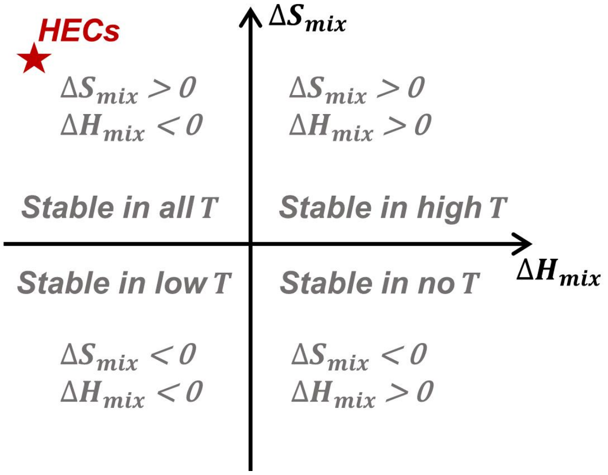

Fig. S11. Thermodynamic stable phase diagram of HEC. Due to $\Delta S_{mix} < 0$ and $\Delta H_{mix} < 0$, HEC can be form as a stable single phase at all temperatures, which is revealed to be an entropy-driven structural stabilization effect(*32*).

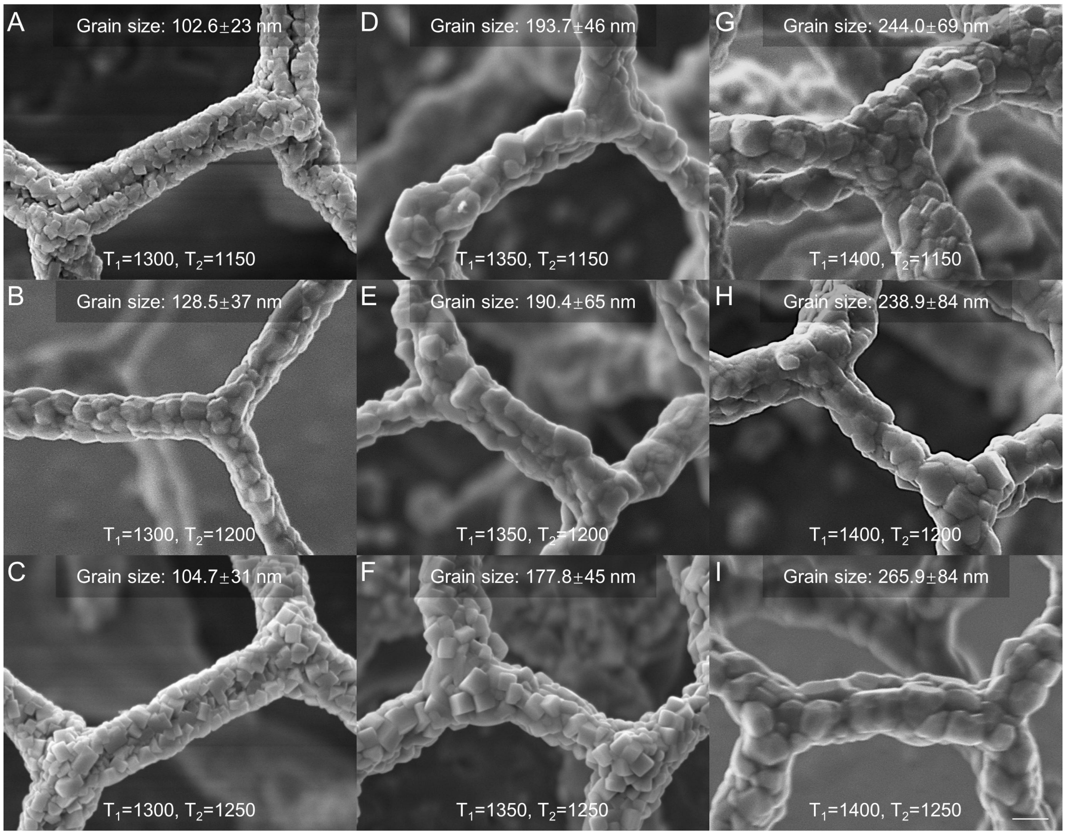

Fig. S12. SEM images of HEC after two-step sintering with different temperatures to reveal grain evolution. Scale bars: 400 nm. Data are presented as mean ± s.d. Data in (**A** to **I**) are from n = 3 independent samples.

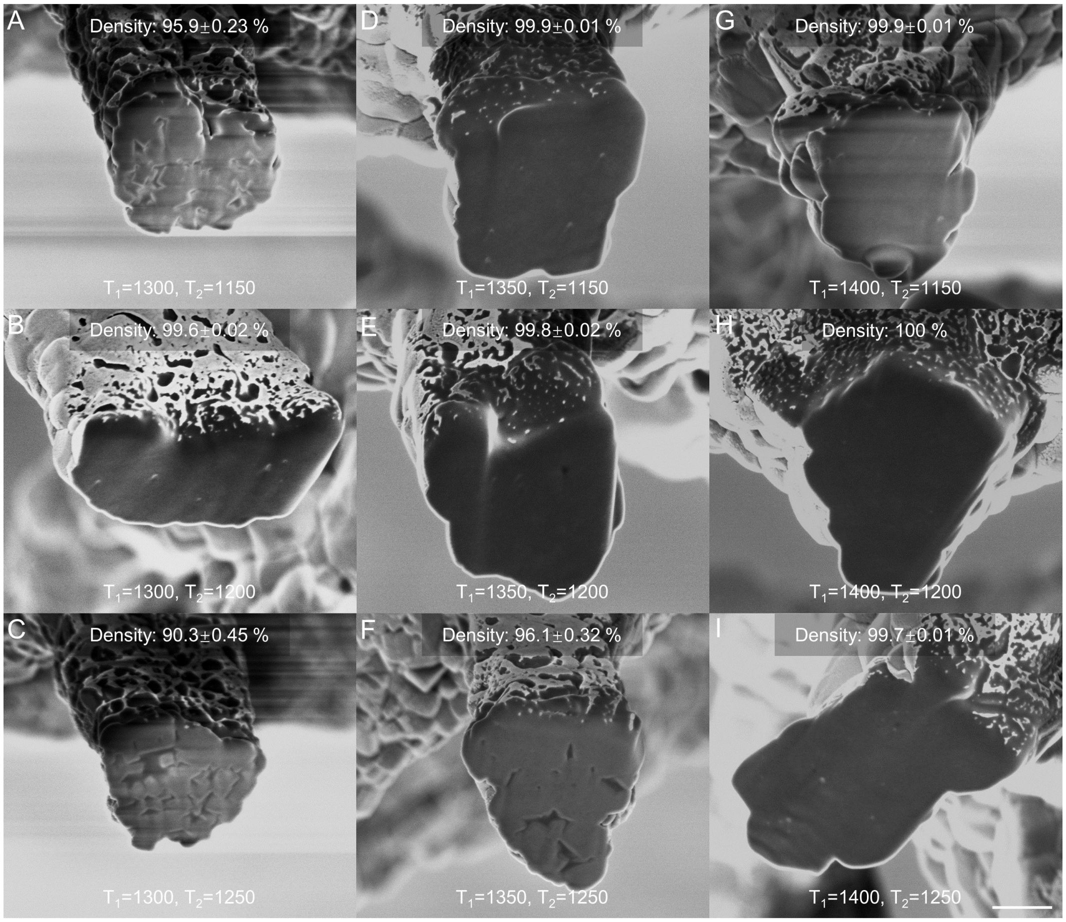

Fig. S13. SEM images of FIB-cut HEC after two-step sintering with different temperatures to reveal densification evolution. Scale bars: 200 nm. Data are presented as mean ± s.d. Data in (**A** to **I**) are from n = 3 independent samples.

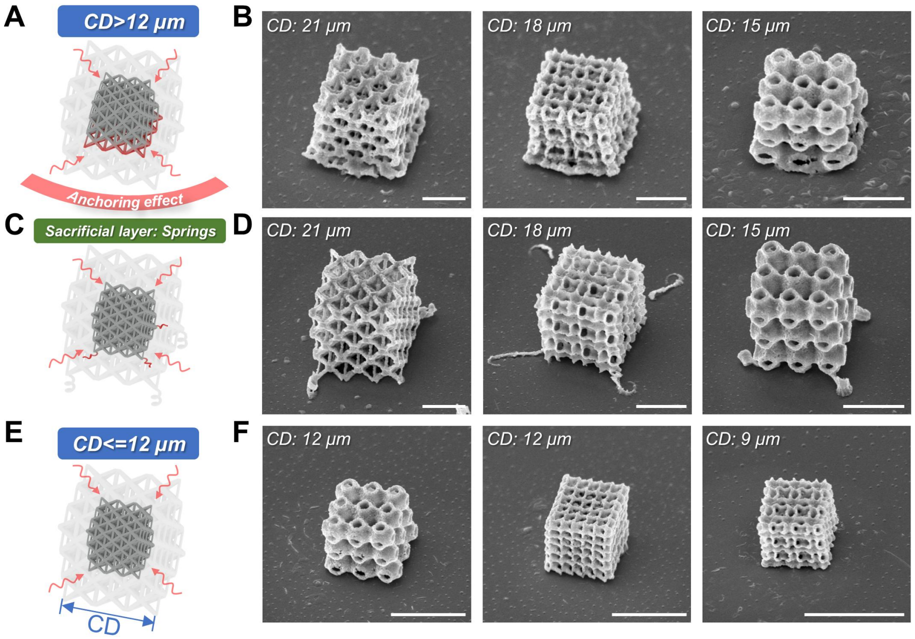

Fig. S14. Anchoring effect dependent on critical dimension (CD) during sintering. (**A**) Illustration of anchoring effect. (**B**) SEM images exhibit HEC structures with a deformed base because of the adhesion between the bottom layer and the substrate when CD is over 12 μm. To circumvent this issue, sacrificial support structures (springs) have been used to support the main structures (**C**). (**D**) SEM images of HEC nanolattices with uniform shrinkage and high-fidelity, demonstrating the effectiveness of the springs sacrificial layer. (**E**) Illustration of disappearance of anchoring effect when CD is reduced to 12 μm. (**F**) SEM images of HEC nanolattices with CD of 12 μm and 9 μm. All structures show the successful uniform heat-shrinking of three-dimensional prints without any sacrificial layer. Scale bars: 5 μm.

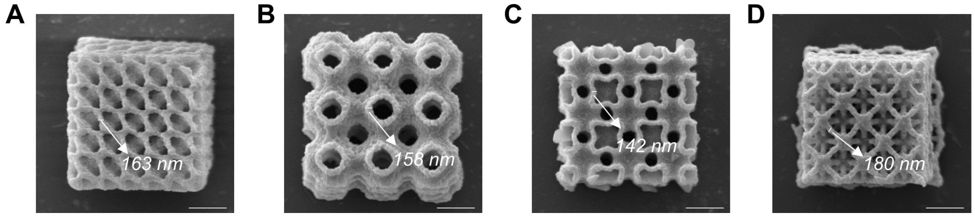

Fig. S15. Nanolattices of HEC with feature size down to 150nm. (**A**) Diamond nanolattice. (**B**) Schwarz P nanolattice. (**C**) I-WP nanolattice. (**D**) Octet nanolattice. Scale bars: 1 μm. The obtained high surface quality with small wall thickness is achieved by collaborative contribution from optimized laser power, "writing direction down" method and two-step sintering strategy (more detail in "Materials and Methods" section).

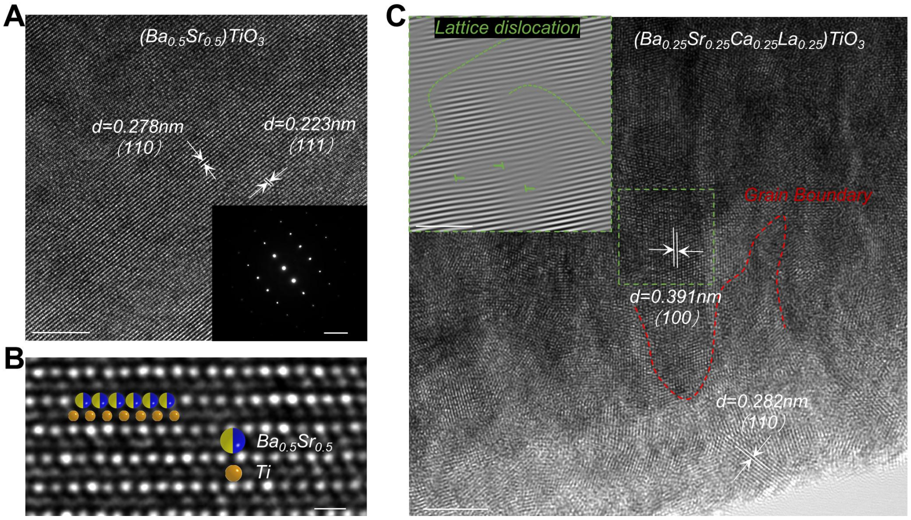

Fig. S16. Microscopic structure of $(Ba_{0.5}Sr_{0.5})TiO_3$ and $(Ba_{0.25}Sr_{0.25}Ca_{0.25}La_{0.25})TiO_3$. (**A**) HRTEM images with SAED in the bottom right corner of the $(Ba_{0.5}Sr_{0.5})TiO_3$. The perovskite structure with clear lattice spacings of 0.278 and 0.223 nm can be examined in the HRTEM image which are associated with the (110) and (111) facets continues to exist stably. Scale bars: 5 nm and 5 nm^{-1}. (**B**) The atomic-resolution HRTEM image of $(Ba_{0.5}Sr_{0.5})TiO_3$ directly illustrates the arranged atoms with various brightness. The inset depicts the schematic representation of the lattice structure of perovskite phase, with Sr, Ba occupying the A sites and Ti occupying the B sites, separately. Scale bar: 0.5 nm. (**C**) HRTEM image of $(Ba_{0.25}Sr_{0.25}Ca_{0.25}La_{0.25})TiO_3$ ceramic. The inset, the inverse fast Fourier transform (IFFT) filtered image deduced from the green box area in (**C**), displays some lattice dislocations. Scale bars: 5 nm and 2 nm (inset).

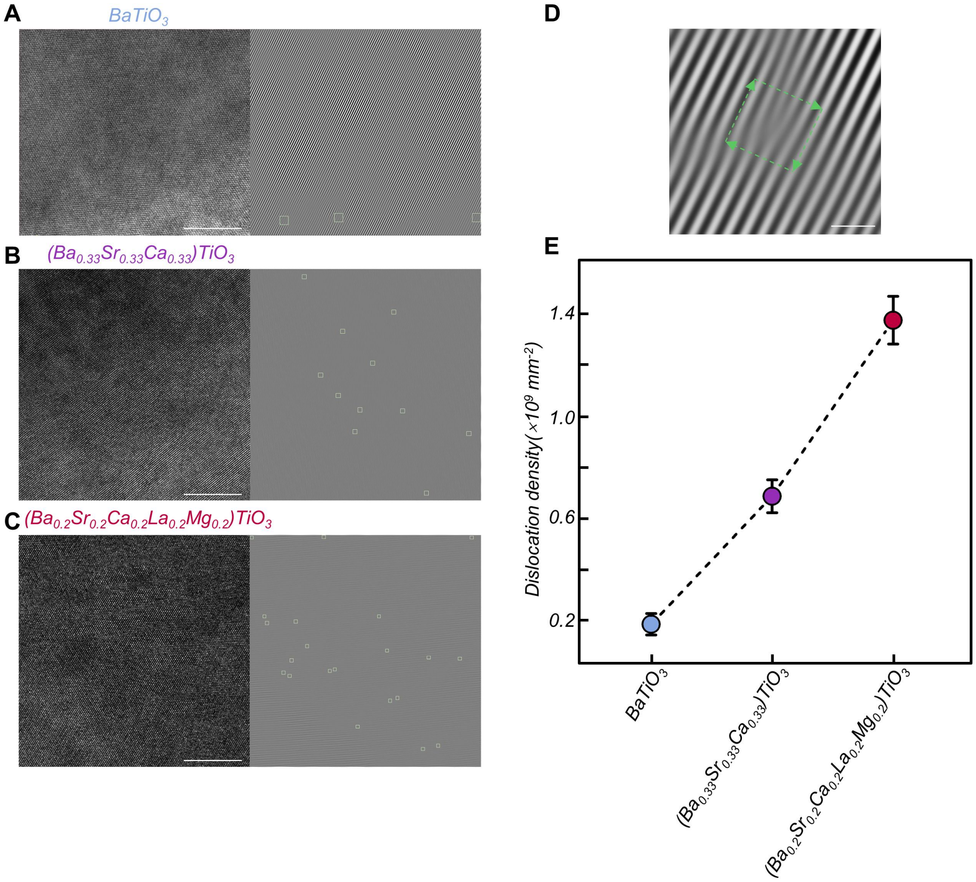

Fig. S17. Locating dislocations in HRTEM images and Calculation of dislocation density. HRTEM images and corresponding inverse fast Fourier transform (IFFT) filtered images of (**A**) $BaTiO_3$, (**B**) $(Ba_{0.33}Sr_{0.33}Ca_{0.33})TiO_3$, (**c**) $(Ba_{0.2}Sr_{0.2}Ca_{0.2}La_{0.2}Mg_{0.2})TiO_3$. The dislocations are marked by light green squares. Scale bars: 20 nm. (**D**) The IFFT was used to assist the HRTEM image to fix the position of dislocations. The existence of dislocation was verified by drawing a Burgers circuit. Scale bar: 1 nm. (**E**) Dislocation density varies with increasing principle elements . Data are presented as mean ± s.d. Data in (D) are from n = 3 independent samples.

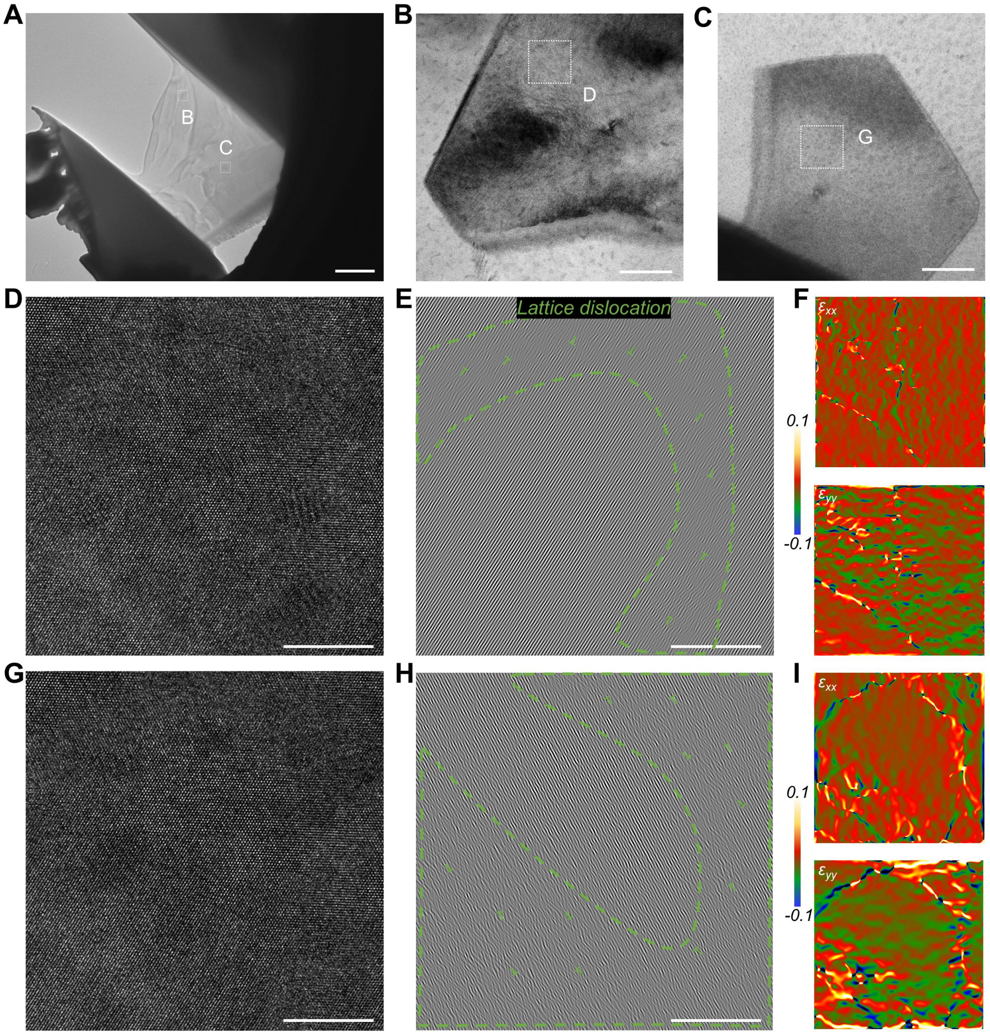

Fig. S18. Morphology and microstructure of HEC after High temperature annealing (1400 °C) to reveal the stability of dislocations. (A) TEM sample image at a low magnification. Scale bar: 1 µm. (**B** and **C**) Two enlarged image from (**A**). Scale bars: 50 nm. (**D** and **G**) HRTEM images with clear lattice and (**E** and **H**) the corresponding inverse fast Fourier transform filtered patterns of HEC. Scale bars:10 nm. (**F** and **I**) Strain distribution images along the ε_{xx} and ε_{yy} directions of (**D** and **G**), respectively.

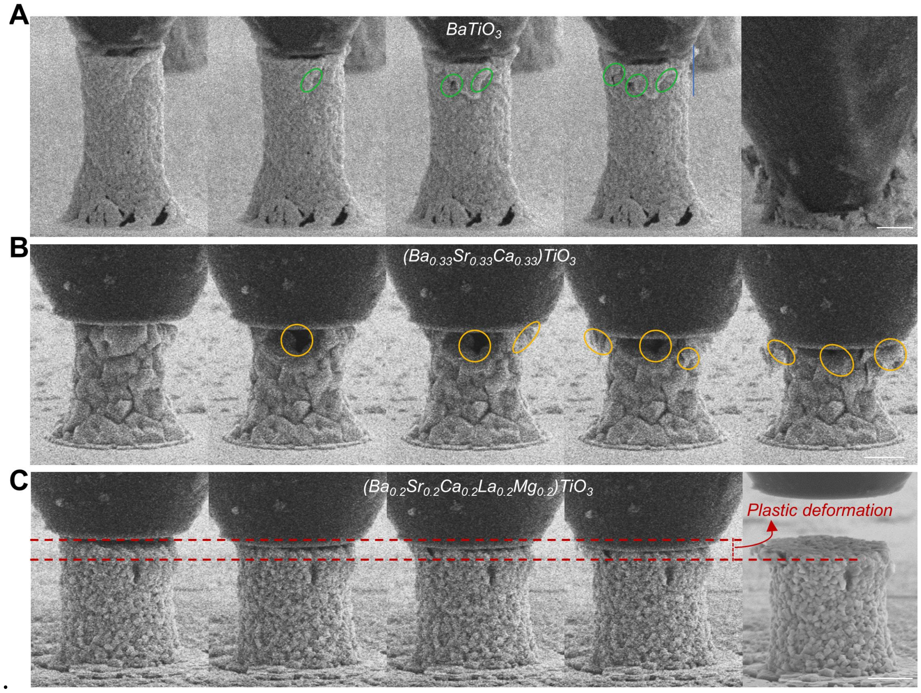

Fig. S19. Comparison of ceramics with different entropies when strain increasing. In-situ SEM compression recorded video screenshot of pillars of low-entropy $BaTiO_3$ (**A**), medium-entropy $(Ba_{0.33}Sr_{0.33}Ca_{0.33})TiO_3$ (**B**), high-entropy $(Ba_{0.2}Sr_{0.2}Ca_{0.2}La_{0.2}Mg_{0.2})TiO_3$ (**C**). In low entropy and medium entropy systems, cracks and ceramic fragments are the mediums to release stress. HEC cylinder bears greater compressive stress through significant plastic deformation. Scale bars: 4 μm (**A**) and 3 μm (**B** and **C**).

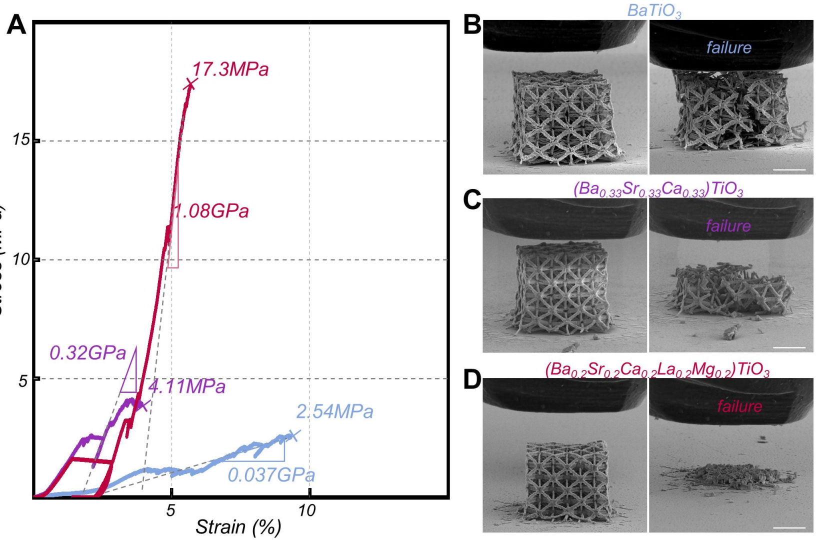

Fig. S20. Mechanical behavior comparison of 3D nanostructure ceramics with different entropies. (**A**) Stress–strain curves of octet ceramic nanolattices with different entropies. The differences in mechanical behavior and properties of nanolattices stem from their constituents. (**B** to **D**) The corresponding snapshots of nanolattices before and after in-situ compression experiment. Scale bars: 10 μm.

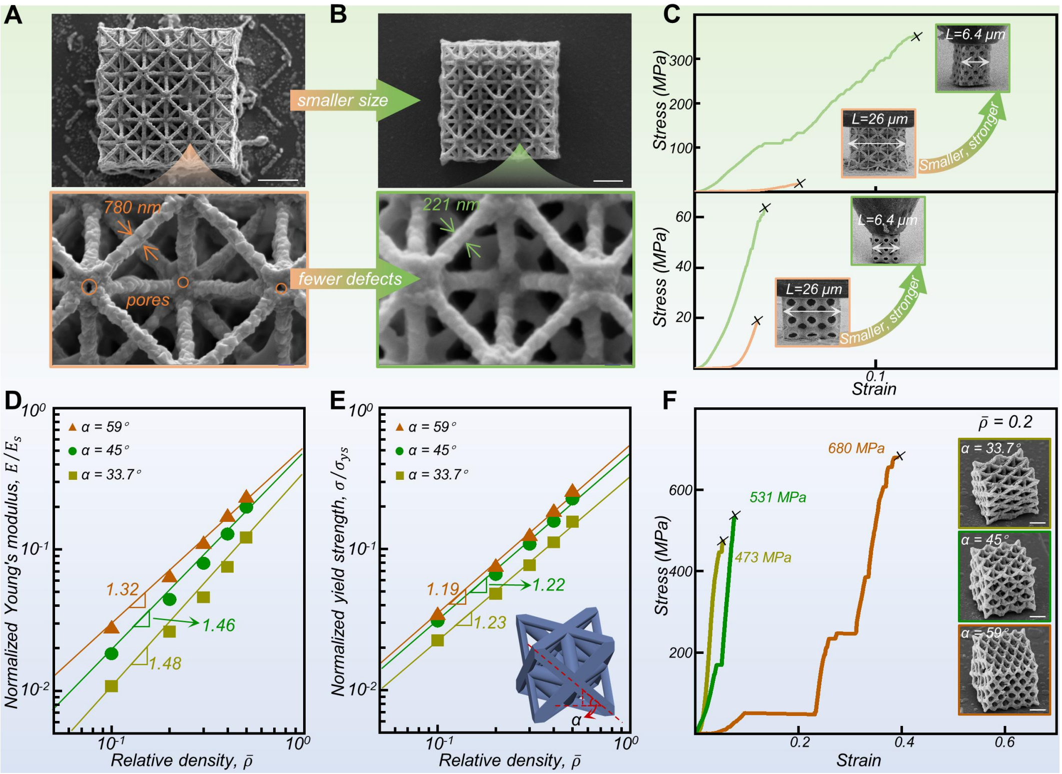

Fig. S21. Size effect and structural design related to power law scaling with relative density. (**A** and **B**) SEM images of HEC large-scale and small-scale octet nanolattices. Smaller size makes fewer defects. This difference originates from the fact that as the material scale decreases, defects that follow the statistical distribution gradually become difficult to observe. Scale bars: 10 μm and 2 μm, respectively. (**C**) Stress–strain curves of HEC octet and shell-based nanolattices with large size and small size, indicating the strength of small-scale nanolattices is higher than that of their large-scale counterparts and demonstrating the size strengthening effects also known as "smaller and stronger"(*23*). (**D**) Log–log plots of the normalized Young's modulus vs. the relative density of different unit cells. (**E**) Log–log plots of the normalized yield strength vs. the relative density of different unit cells. (**F**) Stress–strain curves of HEC octet nanolattices with different characteristic angles at a relative density of 0.2, respectively. These results imply that an octet-truss with a larger characteristic angle is more approaching an ideal stretching dominated structure. Scale bars: 2 μm.

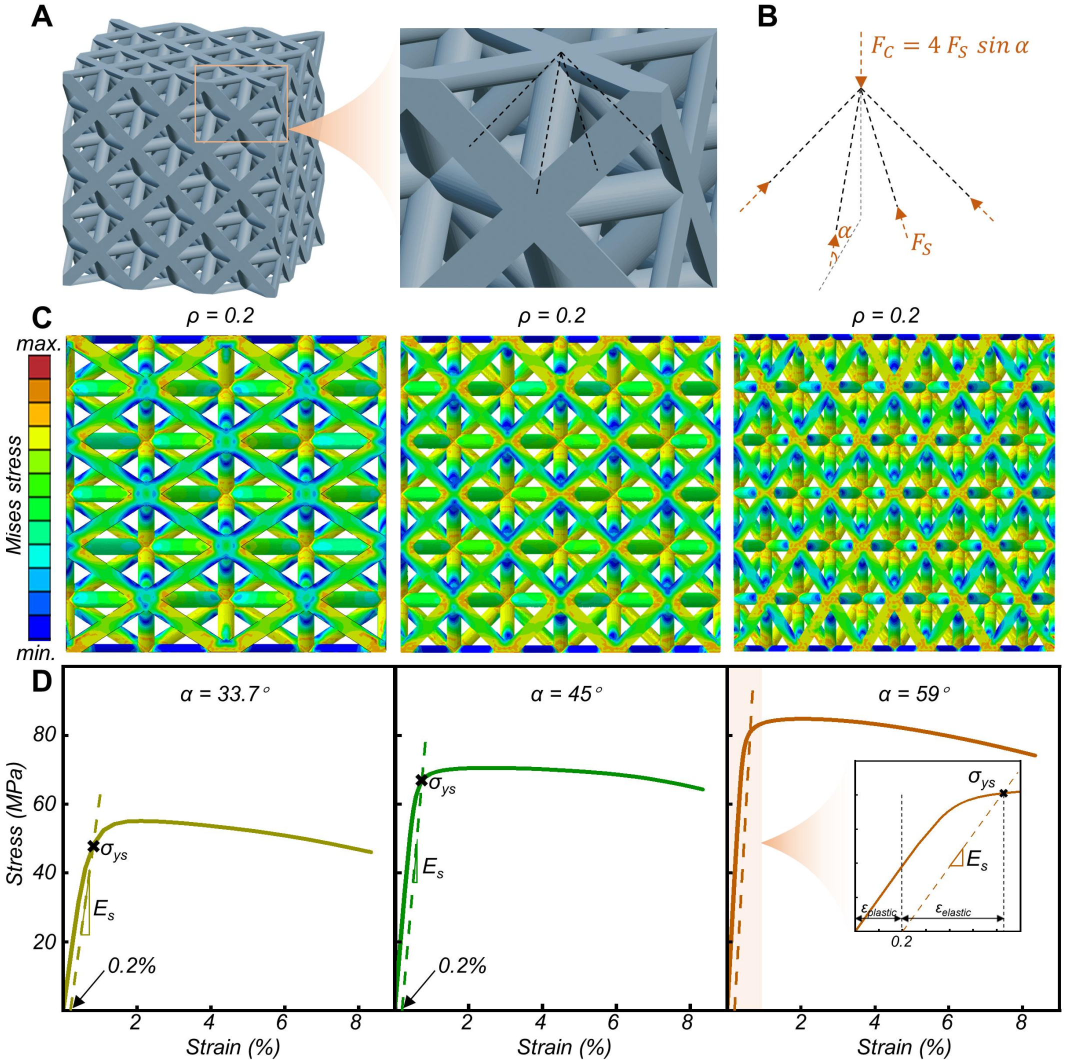

Fig. S22. Mechanical properties FEM for octet nanolattices with different characteristic angles. (**A** and **B**) Geometric relations in the octet lattice cell. Assuming an ideally pin-jointed framework, the load at failure in a single lattice strut, F_S, can be estimated applying the measured maximum load, F_C, and the trigonometric relations shown. A larger α increases the compressive force along the beam and reduces bending stress. (**C**) Von Mises stress distribution of octet nanolattices with different α at a relative density of ~20% under the 0.2% offset yield point. (**D**) Stress-strain curves of octet nanolattices with different α at a relative density of ~20%. The lattice's Young's modulus is calculated using the maximal slope of the linear regime of the stress-strain curve and the yield strength is extracted from the 0.2% yield offset of the curve. The inset displays the plastic and elastic deformation of the structure.

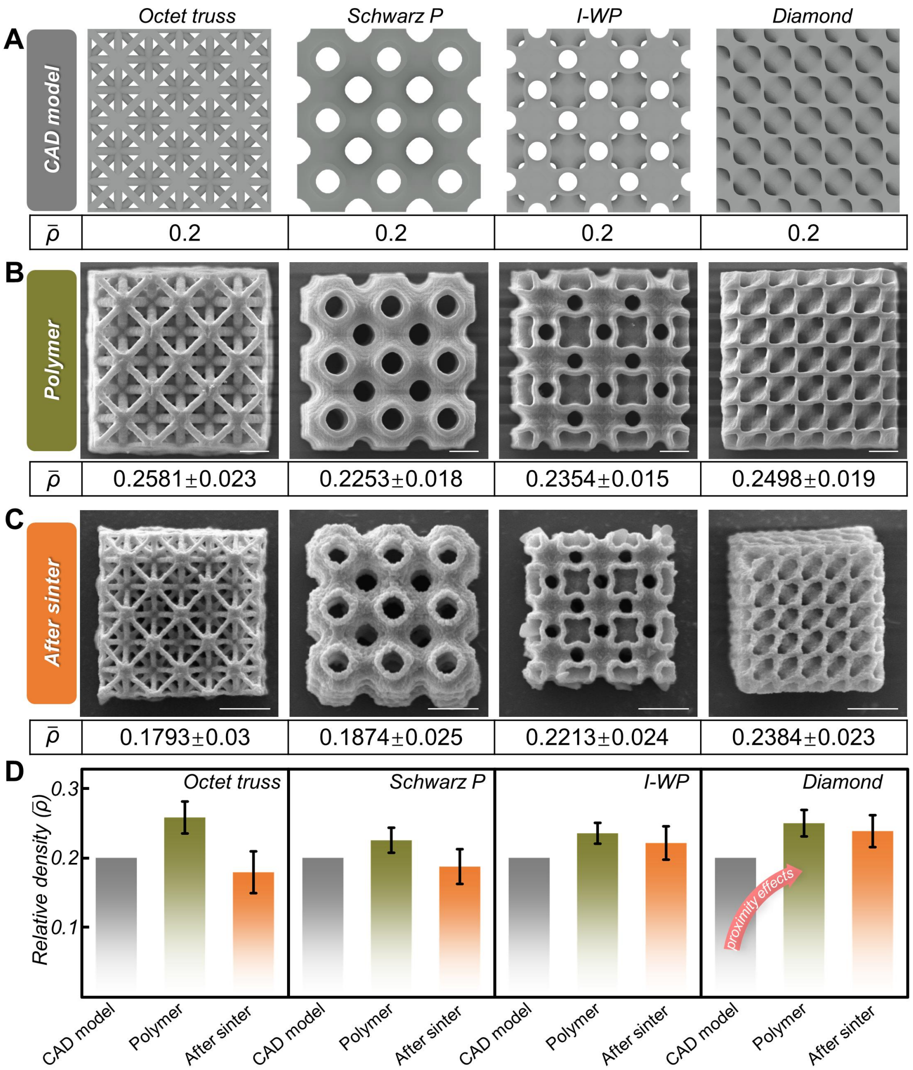

Fig. S23. Structure relative density variation during the fabricating process. (A) CAD models, (**B**) polymer SEM images, (**C**) sintered HEC SEM images and (**D**) statistical data of relative densities of different structures. Scale bars: 2 μm. The relative density of sintered HEC structures is recalculated by adjusting the beam or wall thickness in the original CAD mode with the SEM-measured sizes. It is evident that the relative density of polymer is greater than the relative density of corresponding CAD model due to proximity effects in two-photon lithography(*76*). And after sintering, the relative density of structure generally decreases because of ceramic material's crystallization and densification. Data are presented as mean ± s.d. Data in (**B** and **C**) are from n = 5 independent samples.

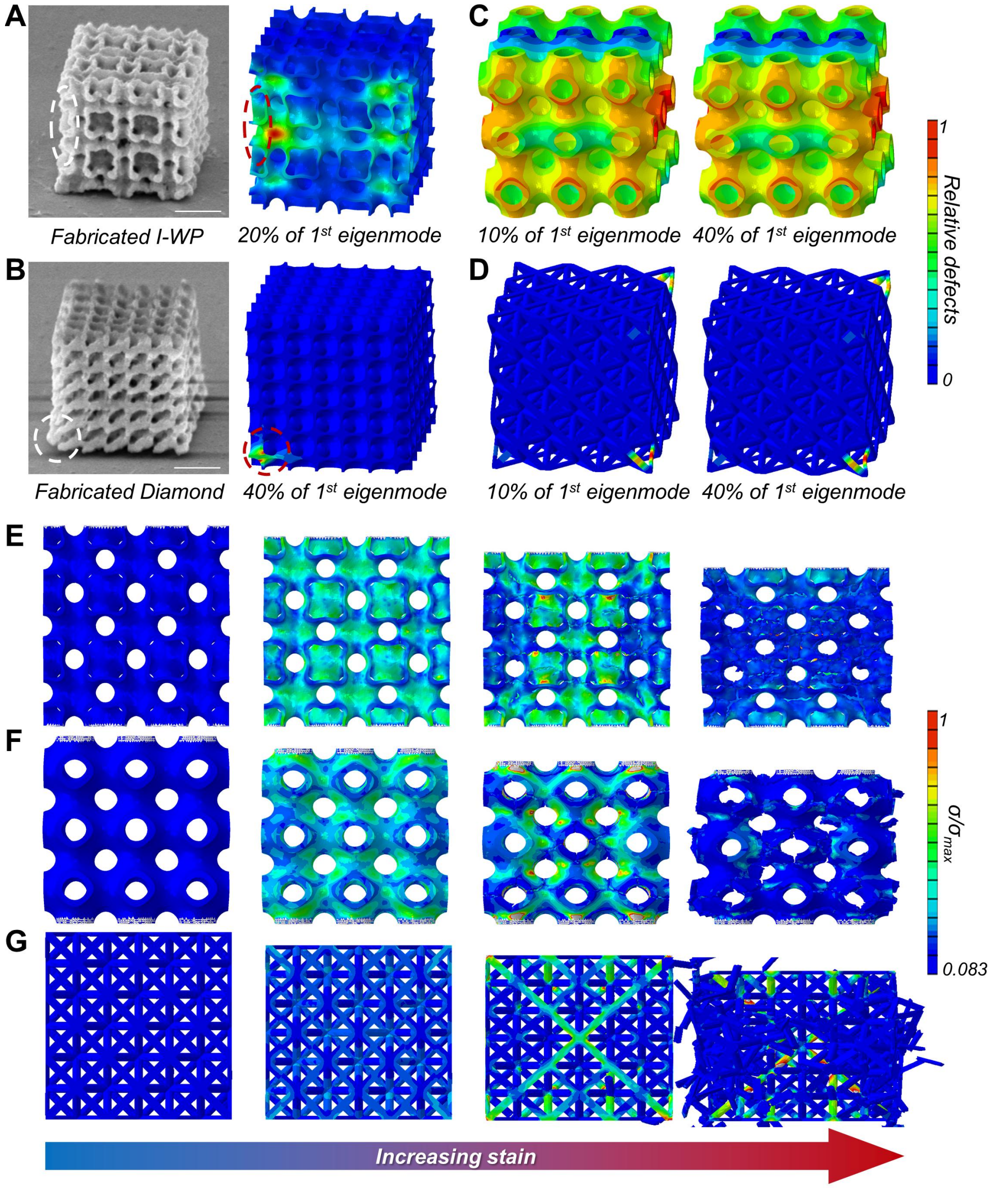

Fig. S24. FE simulations of HEC compressing samples with different cells. Simulated configurations of I-WP (**A**), Diamond (**B**), Schwarz P (**C**) and octet-truss (**D**) nanolattices with preexisting defects introduced by imposing 0, 10%, 20%, 40% 1st eigenmode. Scale bars: 2 μm. The SEM images of fabricated nanolattices display that some parts of structures are pre-bent or distorted, which are very similar to corresponding FE models. The color represents the extent of initial defects. Snapshots from finite element modelling of I-WP (**E**), Schwarz P (**F**), and octet-truss (**G**) at different strains.

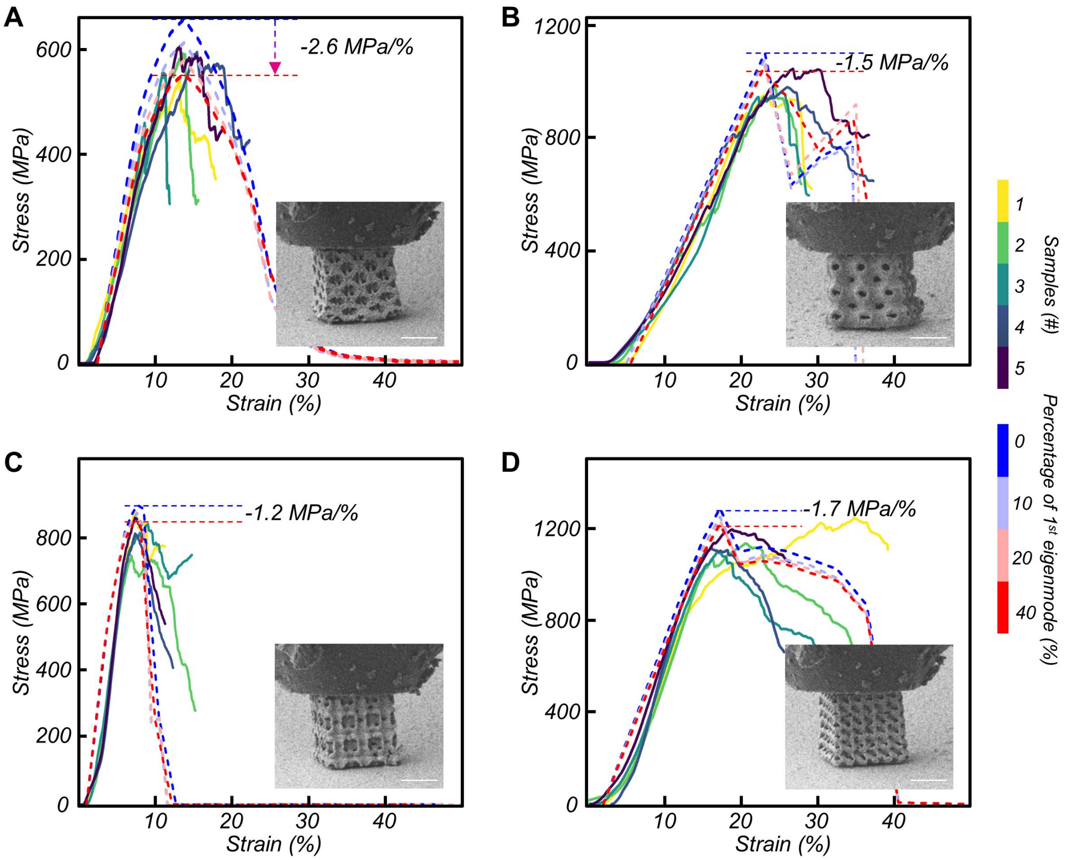

Fig. S25. Compressive stress-strain curves from simulations and experiments. (**A**) Compressive stress-strain curves of octet-truss. (**B**) Compressive stress-strain curves of Schwarz P. (**C**) Compressive stress-strain curves of I-WP. (**D**) Compressive stress-strain curves of Diamond. Scale bars: 3 μm. All stress-strain curves from simulations are applied with different percentages of 1st eigenmode as initial structural defects. It is that found that the initial defects of structures can reduce the compressive strength of nanolattices. Specifically, the relative reduction of octet-truss is 2.6 MPa/%, the relative reduction of Schwarz P is 1.5 MPa/%, the relative reduction of I-WP is 1.2 MPa/% and the relative reduction of Diamond is 2.6 MPa/%.

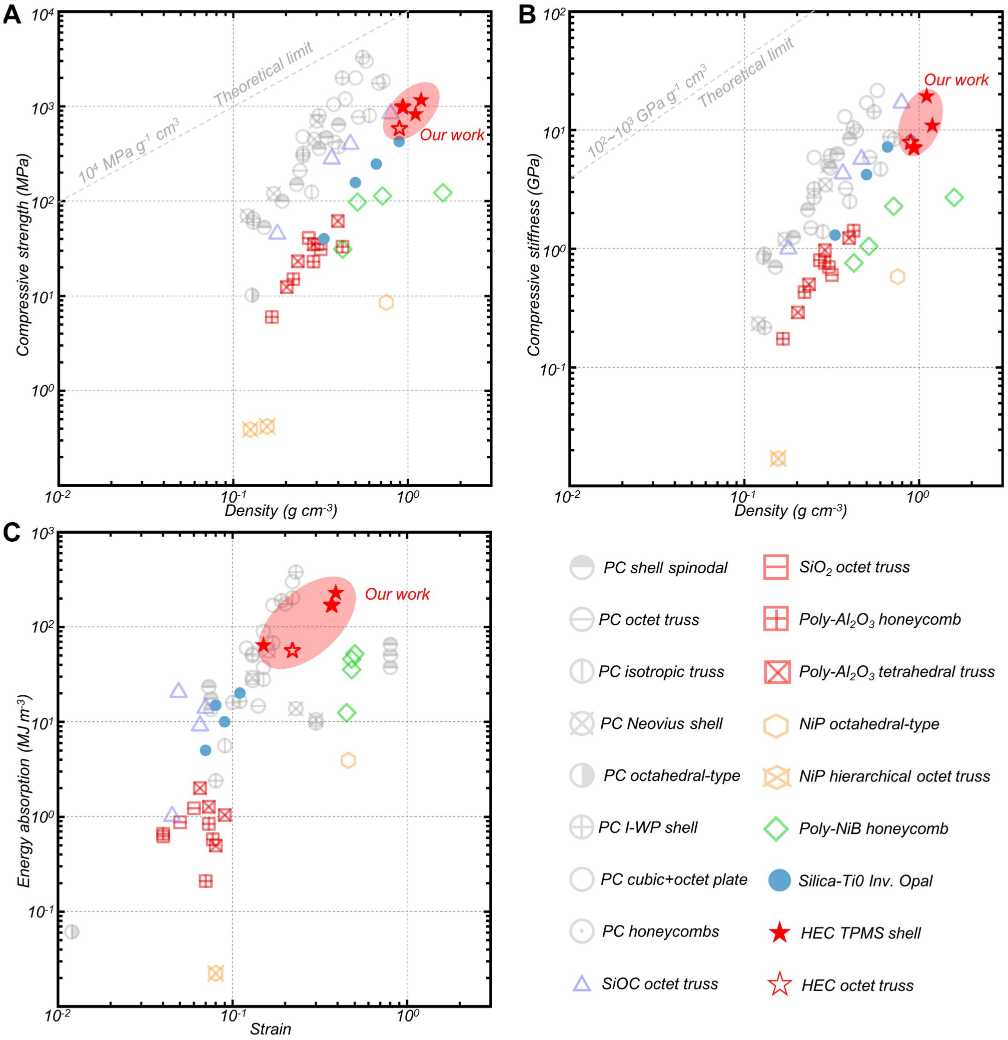

Fig. S26. Mechanical property comparison of advanced micro/nano lattices reported to date(*13, 16, 49-51, 77-87*). (A) Compressive strength and **(B)** compressive stiffness Ashby maps against density. **(C)** Ashby plot of the energy absorption against strain. The theoretical limit is indicated as an average value with the lower bound defined by diamond (the strongest and stiffest bulk material at the macroscale) and the upper bound given by the strength of CNTs and graphene (the strongest and stiffest known material, albeit in two dimensions and at the nanoscale)(*49, 81*).

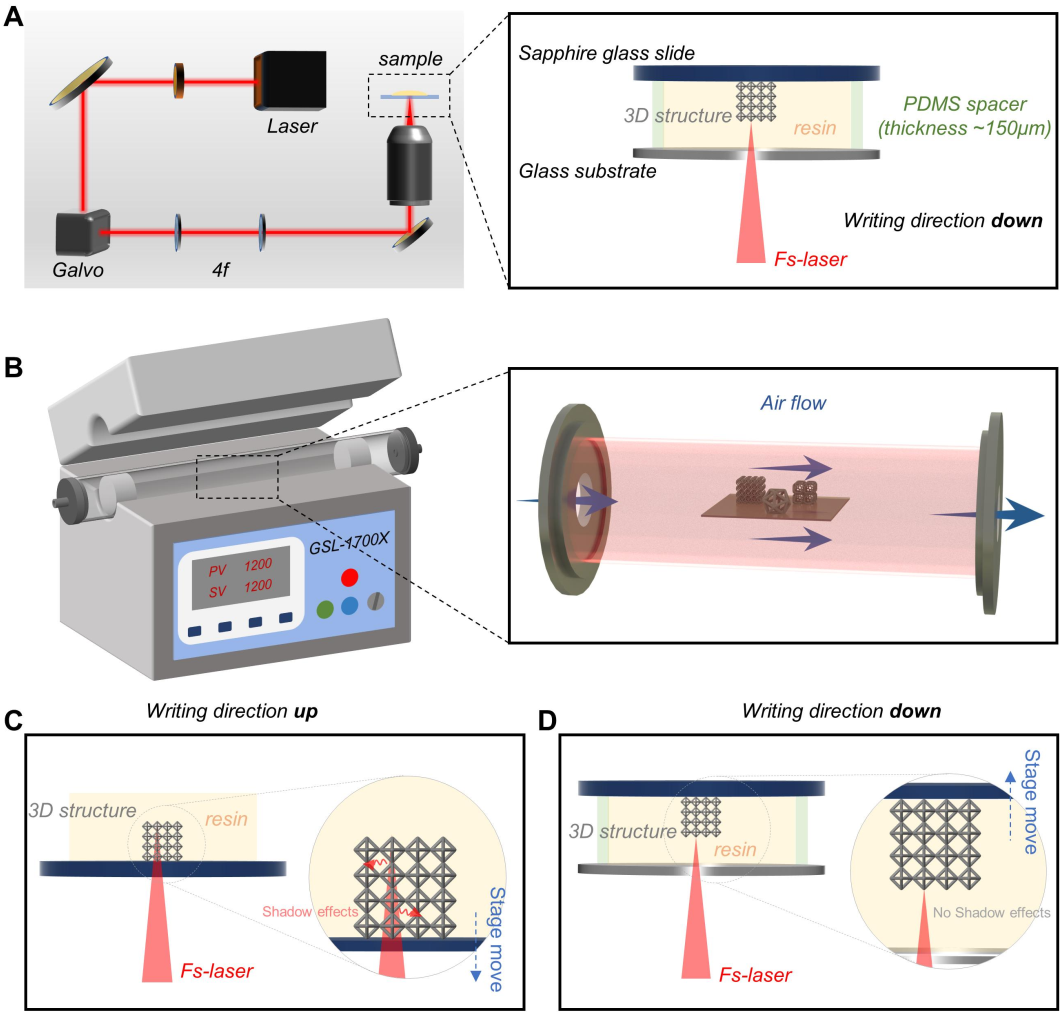

Fig. S27. Schematic diagram of TPP and sintering setup for HEC. Schematic illustration of the two-photon nano-printing setup (**A**) and sintering furnace device (**B**). In fact, the standard printing approach of 2PP (non-immersion mode), also named as "writing direction up" (C), frequently encounters the drawbacks of the focus distortion and laser power loss, due to shadowing effects when the laser passes through already solidified volumes to move the focus at decreasing z-coordinates of stage(*88*). This is especially common when nonstandard resist materials are used, as the customized HEC resin. In contrast, this "writing direction down" method (D) enables to avoid shadowing effects, and assured a constant exposure dose because the laser only passe through just-polymerized layers when increasing z-coordinates of stage. Because of this, it was possible to maintain a high fabrication resolution from the bottom to the top of the 3D structure, as the focus was not going through the solidified volumes.

	$\Delta S_{mix}(R)$	ΔG_{mix} (10^{-2} eV/atom)	$t_{effective}$	δ(%)
$BaTiO_3$	0	0	0.9676	0
$(Ba_{0.5}Sr_{0.5})TiO_3$	0.69	-1.486	0.9289	1.8
$(Ba_{0.33}Sr_{0.33}Ca_{0.33})TiO_3$	1.1	-2.315	0.8996	5.37
$(Ba_{0.25}Sr_{0.25}Ca_{0.25}La_{0.25})TiO_3$	1.39	-7	0.8911	4.65
$(Ba_{0.2}Sr_{0.2}Ca_{0.2}La_{0.2}Mg_{0.2})TiO_3$	1.61	-5.967	0.8663	12.5

Table S1. Theoretical design parameters for HEC. The configurational entropy, the Gibbs free energy, the Goldschmidt tolerance factor and the Lattice size difference of ceramics with different numbers of metals in A site. Although the significantly distorted lattice from the unbalance among ionic size, mass, and bond state, HEC may still exist stably due to the entropy-dominated phase stabilization effect and a negative ΔG_{mix}.

Ceramic	*crosslinker*	*solvent*	*Solid fraction (wt%)*	*Precursor types*
SiO_2	*PEGDA, TET*	*PGMEA*	*8.8*	*1*
SiOC	*TMSPM*	*THF, DOWANOL TPM*	*33.4*	*1*
ZrO_2	*PETA*	*DMAc, DCM*	*2.4*	*1*
TiO_2	*PETA*	*2-methoxyethanol, DCM*	*28.3*	*1*
ZnO	*PEGda*	*Water, DMSO*	*35.4*	*1*
$BaZrO_3$	*PETA*	*MeOH, DCM, DMSO*	*3.6*	*3*
SnO_2	*PETA*	*2-methoxyethano*	*13.7*	*1*
$Y_3Al_5O_{12}$	*AAC*	*ethylene glycol, TDW*	*25*	*1*
HEC	*None*	*None*	*13.5*	*15+*

Table S2. Contrast of particle free organic-inorganic photoresists containing metal salts in previous works(*16-19, 21, 33, 34, 89*).

Structure	*Relative density($\bar{\rho}$)*	*Density (ρ, g cm^{-3})*	*Strength (MPa)*	*Stiffness (GPa)*	*Energy absorption (MJ m^{-3})*
Octet truss	*0.1793±0.03*	*0.8929±0.15*	*580.6±24.9*	*7.03±1.2*	*56.35±17.47*
Schwarz P	*0.1874±0.025*	*0.9332±0.12*	*983.4±32*	*6.628±0.87*	*63.86±10.9*
I-WP	*0.2213±0.024*	*1.102±0.12*	*827.2±47*	*21.25±4.02*	*169.24±38.56*
Diamond	*0.2384±0.023*	*1.187±0.115*	*1159.2±54.5*	*12.96±2.6*	*277.86±57.8*

Table S3. Numerical results of all tested HEC structures. Data are presented as mean ± s.d. Data are from n = 3 independent samples.

	Ba	*Sr*	*Ca*	*La*	*Mg*	*Ti*	*O*
ionic radius(pm)	*135*	*113*	*99*	*106.1 (3+)*	*65*	*68 (4+)*	*140 (2-)*
atomic radius(pm)	*198*	*191*	*174*	*187.7*	*136*	*132*	*66*

Table S4. Atomic and ionic radii of elements used in HEC.

Metal acrylates (1 mol)	Raw material (mol)	Step 1: synthesis		Step 2: dissolve	
		AAC	H_2O	AAC	H_2O
Ti-AAC	$Ti(C_2H_5O)_4$ (1)	4 mol			
Ba-AAC	$Ba(OH)_2$ (1)	2 mol	2 mol	4 mol	4 mol
Sr-AAC	$Sr(OH)_2$ (1)	2 mol	2 mol	3 mol	3 mol
Ca-AAC	$Ca(OH)_2$ (1)	2 mol	2 mol	3 mol	3 mol
La-AAC	La_2O_3 (0.5)	3 mol	2 mol	5 mol	5 mol
Mg-AAC	$Mg(OH)_2$ (1)	2 mol	2 mol	4 mol	4 mol
Li-AAC	LiOH (1)	1 mol	2 mol	2 mol	2 mol
K-AAC	KOH (1)	1 mol	2 mol	3 mol	3 mol
Na-AAC	NaOH (1)	1 mol	2 mol	3 mol	3 mol
Zn-AAC	$Zn(OH)_2$ (1)	2 mol	2 mol	4 mol	4 mol
Pb-AAC	$2PbCO_3 \cdot Pb(OH)_2$ (0.33)	2 mol	2 mol	5 mol	5 mol
Nd-AAC	Nd_2O_3 (0.5)	3 mol	2 mol	7 mol	7 mol
Sm-AAC	Sm_2O_3 (0.5)	3 mol	2 mol	6 mol	6 mol
Eu-AAC	Eu_2O_3 (0.5)	3 mol	2 mol	6 mol	6 mol
Nb-AAC	$Nb(C_2H_5O)_5$ (1)	5 mol			
Sn-AAC	$Sn(C_2H_5O)_2$ (1)	2 mol			
ln-AAC	$In(OH)_3$ (1)	3 mol	2 mol	5 mol	5 mol

Table S5. Material ratio in the preparation process of different metal acrylates.

Compression behavior			*Tension behavior*		
σ_c *(MPa)*	$\varepsilon_{c,in}$	d_c	σ_t *(MPa)*	$\varepsilon_{t,in}$	d_t
841.9243986	*0*	*0*	*123.7113402*	*0*	*0*
1000	*0.001115589*	*0*	*101.0309278*	*0.000301633*	*0.183324486*
914.0893471	*0.002978855*	*0.085910653*	*77.31958763*	*0.00073425*	*0.374993229*
766.3230241	*0.005031671*	*0.233676976*	*66.66666667*	*0.000965078*	*0.461105273*
632.3024055	*0.007042366*	*0.367697595*	*54.98281787*	*0.001198889*	*0.555550741*
518.9003436	*0.008989876*	*0.481099656*	*37.11340206*	*0.0018506*	*0.69999675*
436.4261168	*0.010842611*	*0.563573883*	*25.08591065*	*0.002285405*	*0.797220025*
384.8797251	*0.01220057*	*0.615120275*	*17.18213058*	*0.010308278*	*0.861109606*
257.7319588	*0.016370203*	*0.742268041*			
171.8213058	*0.020633469*	*0.828178694*			

Table S6. Material damage behaviors used in FE simulation.

Movie S1.
Compress tests of ceramic pillars with different entropies.

Movie S2.
Compress tests of HEC and pyrolytic carbon octet nanolattices.

Movie S3.
Compress tests of TPMS nanolattices of HEC.

REFERENCES AND NOTES

1. X. Zheng, H. Lee, T. H. Weisgraber, M. Shusteff, J. DeOtte, E. B. Duoss, J. D. Kuntz, M. M. Biener, Q. Ge, J. A. Jackson, S. O. Kucheyev, N. X. Fang, C. M. Spadaccini, Ultralight, ultrastiff mechanical metamaterials. *Science* **344**, 1373–1377 (2014).

2. M. A. Saccone, R. A. Gallivan, K. Narita, D. W. Yee, J. R. Greer, Additive manufacturing of micro-architected metals via hydrogel infusion. *Nature* **612**, 685–690 (2022).

3. F. Kotz, K. Arnold, W. Bauer, D. Schild, N. Keller, K. Sachsenheimer, T. M. Nargang, C. Richter, D. Helmer, B. E. Rapp, Three-dimensional printing of transparent fused silica glass. *Nature* **544**, 337–339 (2017).

4. Y.-L. Zhang, Q.-D. Chen, H. Xia, H.-B. Sun, Designable 3D nanofabrication by femtosecond laser direct writing. *Nano Today* **5**, 435–448 (2010).

5. J. B. Berger, H. N. G. Wadley, R. M. McMeeking, Mechanical metamaterials at the theoretical limit of isotropic elastic stiffness. *Nature* **543**, 533–537 (2017).

6. D. Jang, J. R. Greer, Transition from a strong-yet-brittle to a stronger-and-ductile state by size reduction of metallic glasses. *Nat. Mater.* **9**, 215–219 (2010).

7. S. K. Saha, D. Wang, V. H. Nguyen, Y. Chang, J. S. Oakdale, S.-C. Chen, Scalable submicrometer additive manufacturing. *Science* **366**, 105–109 (2019).

8. V. Hahn, P. Rietz, F. Hermann, P. Müller, C. Barner-Kowollik, T. Schlöder, W. Wenzel, E. Blasco, M. Wegener, Light-sheet 3D microprinting via two-colour two-step absorption. *Nat. Photonics* **16**, 784–791 (2022).

9. W. Ouyang, X. Xu, W. Lu, N. Zhao, F. Han, S.-C. Chen, Ultrafast 3D nanofabrication via digital holography. *Nat. Commun.* **14**, 1716 (2023).

10. L. R. Meza, A. J. Zelhofer, N. Clarke, A. J. Mateos, D. M. Kochmann, J. R. Greer, Resilient 3D hierarchical architected metamaterials. *Proc. Natl. Acad. Sci. U.S.A.* **112**, 11502–11507 (2015).

11. T. Tancogne-Dejean, M. Diamantopoulou, M. B. Gorji, C. Bonatti, D. Mohr, 3D plate-lattices: An emerging class of low-density metamaterial exhibiting optimal isotropic stiffness. *Adv. Mater.* **30**, e1803334 (2018).

12. L. R. Meza, S. Das, J. R. Greer, Strong, lightweight, and recoverable three-dimensional ceramic nanolattices. *Science* **345**, 1322–1326 (2014).

13. J. Bauer, S. Hengsbach, I. Tesari, R. Schwaiger, O. Kraft, High-strength cellular ceramic composites with 3D microarchitecture. *Proc. Natl. Acad. Sci. U.S.A.* **111**, 2453–2458 (2014).

14. D. Jang, L. R. Meza, F. Greer, J. R. Greer, Fabrication and deformation of three-dimensional hollow ceramic nanostructures. *Nat. Mater.* **12**, 893–898 (2013).

15. Z. C. Eckel, C. Zhou, J. H. Martin, A. J. Jacobsen, W. B. Carter, T. A. Schaedler, Additive manufacturing of polymer-derived ceramics. *Science* **351**, 58–62 (2016).

16. Z. Li, Y. Jia, K. Duan, R. Xiao, J. Qiao, S. Liang, S. Wang, J. Chen, H. Wu, Y. Lu, X. Wen, One-photon three-dimensional printed fused silica glass with sub-micron features. *Nat. Commun.* **15**, 2689 (2024).

17. E. Zanchetta, M. Cattaldo, G. Franchin, M. Schwentenwein, J. Homa, G. Brusatin, P. Colombo, Stereolithography of SiOC Ceramic microcomponents. *Adv. Mater.* **28**, 370–376 (2015).

18. D. W. Yee, M. L. Lifson, B. W. Edwards, J. R. Greer, Additive manufacturing of 3D-architected multifunctional metal oxides. *Adv. Mater.* **31**, e1901345 (2019).

19. J. P. Winczewski, J. Arriaga Dávila, M. Herrera-Zaldívar, F. Ruiz-Zepeda, R. M. Córdova-Castro, C. R. Pérez de la Vega, C. Cabriel, I. Izeddin, J. G. E. Gardeniers, A. Susarrey-Arce, 3D-architected alkaline-earth perovskites. *Adv. Mater.* **36**, e2307077 (2023).

20. J. C. Sänger, B. R. Pauw, B. Riechers, A. Zocca, J. Rosalie, R. Maaß, H. Sturm, J. Günster, Entering a new dimension in powder processing for advanced ceramics shaping. *Adv. Mater.* **35**, e2208653 (2022).

21. I. Cooperstein, S. R. K. C. Indukuri, A. Bouketov, U. Levy, S. Magdassi, 3D printing of micrometer-sized transparent ceramics with on-demand optical-gain properties. *Adv. Mater.* **32**, e2001675 (2020).

22. J. Liu, Y. Liu, C. Deng, K. Yu, X. Fan, W. Zhang, Y. Tao, H. Hu, L. Deng, W. Xiong, 3D printing nano-architected semiconductors based on versatile and customizable metal-bound composite photoresins. *Adv. Mater. Technol.* **7**, 2101230 (2021).

23. X. Li, H. Gao, Smaller and stronger. *Nat. Mater.* **15**, 373–374 (2016).

24. C. Oses, C. Toher, S. Curtarolo, High-entropy ceramics. *Nat. Rev. Mater.* **5**, 295–309 (2020).

25. Y. Yang, H. Li, B. Duan, Q. Feng, C. Li, X. Lu, G. Chen, C. Li, A novel high entropy perovskite oxide with co-substitution in A and B sites $(Ca_{1/3}Sr_{1/3}Ba_{1/3})(Y_{1/4}Zr_{1/2}Nb_{1/4})O_3$ design, synthesis and structural characterization. *Ceram. Int.* **49**, 7920–7926 (2023).

26. P. Zhang, L. Gong, Z. Lou, J. Xu, S. Cao, J. Zhu, H. Yan, F. Gao, Reduced lattice thermal conductivity of perovskite-type high-entropy $(Ca_{0.25}Sr_{0.25}Ba_{0.25}RE_{0.25})TiO_3$ ceramics by phonon engineering for thermoelectric applications. *J. Alloy. Compd.* **898**, 162858 (2022).

27. Z. Liu, S. Xu, T. Li, B. Xie, K. Guo, J. Lu, Microstructure and ferroelectric properties of high-entropy perovskite oxides with A-site disorder. *Ceram. Int.* **47**, 33039–33046 (2021).

28. B. Yang, Y. Zhang, H. Pan, W. Si, Q. Zhang, Z. Shen, Y. Yu, S. Lan, F. Meng, Y. Liu, H. Huang, J. He, L. Gu, S. Zhang, L.-Q. Chen, J. Zhu, C.-W. Nan, Y.-H. Lin, High-entropy enhanced capacitive energy storage. *Nat. Mater.* **21**, 1074–1080 (2022).

29. Y. Sun, S. Dai, High-entropy materials for catalysis: A new frontier. *Sci. Adv.* **7**, eabg1600 (2021).

30. S. Jiang, T. Hu, J. Gild, N. Zhou, J. Nie, M. Qin, T. Harrington, K. Vecchio, J. Luo, A new class of high-entropy perovskite oxides. *Scr. Mater.* **142**, 116–120 (2018).

31. Y. Zhang, Y. J. Zhou, J. P. Lin, G. L. Chen, P. K. Liaw, Solid-solution phase formation rules for multi-component alloys. *Adv. Eng. Mater.* **10**, 534–538 (2008).

32. B. Zhao, Z. Yan, Y. Du, L. Rao, G. Chen, Y. Wu, L. Yang, J. Zhang, L. Wu, D. W. Zhang, R. Che, High-entropy enhanced microwave attenuation in titanate perovskites. *Adv. Mater.* **35**, e2210243 (2023).

33. J. Winczewski, M. Herrera, C. Cabriel, I. Izeddin, S. Gabel, B. Merle, A. Susarrey Arce, H. Gardeniers, Additive manufacturing of 3D luminescent $ZrO_2:Eu^{3+}$ architectures. *Adv. Opt. Mater.* **10**, e2102758 (2022).

34. A. Vyatskikh, R. C. Ng, B. Edwards, R. M. Briggs, J. R. Greer, Additive manufacturing of high-refractive-index, nanoarchitected titanium dioxide for 3D dielectric photonic crystals. *Nano Lett.* **20**, 3513–3520 (2020).

35. B. Muraro, N. Husing, G. Kickelbick, U. Schubert, Inorganic−organic hybrid polymers by polymerization of methacrylate- or acrylate-substituted oxotitanium clusters with methyl methacrylate or methacrylic acid. *Chem. Mater.* **14**, 9 (2022).

36. M. J. Mayo, Processing of nanocrystalline ceramics from ultrafine particles. *Int. Mater. Rev.* **41**, 85–115 (2013).

37. I. W. Chen, X. H. Wang, Sintering dense nanocrystalline ceramics without final-stage grain growth. *Nature* **404**, 168–171 (2000).

38. T. Mori, H. Wang, W. Zhang, C. C. Ser, D. Arora, C.-F. Pan, H. Li, J. Niu, M. A. Rahman, T. Mori, H. Koishi, J. K. W. Yang, Pick and place process for uniform shrinking of 3D printed micro- and nano-architected materials. *Nat. Commun.* **14**, 5876 (2023).

39. T. M. Smith, C. A. Kantzos, N. A. Zarkevich, B. J. Harder, M. Heczko, P. R. Gradl, A. C. Thompson, M. J. Mills, T. P. Gabb, J. W. Lawson, A 3D printable alloy designed for extreme environments. *Nature* **617**, 513–518 (2023).

40. B. Yang, Q. Zhang, H. Huang, H. Pan, W. Zhu, F. Meng, S. Lan, Y. Liu, B. Wei, Y. Liu, L. Yang, L. Gu, L.-Q. Chen, C.-W. Nan, Y.-H. Lin, Engineering relaxors by entropy for high energy storage performance. *Nat. Energy* **8**, 956–964 (2023).

41. R. Zhang, *C. Wan*g, P. Zou, R. Lin, L. Ma, L. Yin, T. Li, W. Xu, H. Jia, Q. Li, S. Sainio, K. Kisslinger, S. E. Trask, S. N. Ehrlich, Y. Yang, A. M. Kiss, M. Ge, B. J. Polzin, S. J. Lee, W. Xu, Y. Ren, H. L. Xin, Compositionally complex doping for zero-strain zero-cobalt layered cathodes. *Nature* **610**, 67–73 (2022).

42. X. Qian, D. Han, L. Zheng, J. Chen, M. Tyagi, Q. Li, F. Du, S. Zheng, X. Huang, S. Zhang, J. Shi, H. Huang, X. Shi, J. Chen, H. Qin, J. Bernholc, X. Chen, L. Q. Chen, L. Hong, Q. M. Zhang, High-entropy polymer produces a giant electrocaloric effect at low fields. *Nature* **600**, 664–669 (2021).

43. Y. Oshima, A. Nakamura, K. Matsunaga, Extraordinary plasticity of an inorganic semiconductor in darkness. *Science* **360**, 772–774 (2018).

44. X. Shi, H. Chen, F. Hao, R. Liu, T. Wang, P. Qiu, U. Burkhardt, Y. Grin, L. Chen, Room-temperature ductile inorganic semiconductor. *Nat. Mater.* **17**, 421–426 (2018).

45. Y. Han, X. Liu, Q. Zhang, *M. Huang*, Y. Li, W. Pan, P.-a. Zong, L. Li, Z. Yang, Y. Feng, P. Zhang, *C. Wan*, Ultra-dense dislocations stabilized in high entropy oxide ceramics. *Nat. Commun.* **13**, 2871 (2022).

46. F. Hue, M. Hytch, H. Bender, F. Houdellier, A. Claverie, Direct mapping of strain in a strained silicon transistor by high-resolution electron microscopy. *Phys. Rev. Lett.* **100**, 156602 (2008).

47. C. Lee, Y. Chou, G. Kim, M. C. Gao, K. An, J. Brechtl, C. Zhang, W. Chen, J. D. Poplawsky, G. Song, Y. Ren, Y. C. Chou, P. K. Liaw, Lattice-distortion-enhanced yield strength in a refractory high-entropy alloy. *Adv. Mater.* **32**, e2004029 (2020).

48. Y. Li, X. Liu, P. Zhang, Y. Han, *M. Huang*, *C. Wan*, Theoretical insights into the Peierls plasticity in $SrTiO_3$ ceramics via dislocation remodelling. *Nat. Commun.* **13**, 6925 (2022).

49. C. Crook, J. Bauer, A. Guell Izard, C. Santos de Oliveira, J. Martins de Souza e Silva, J. B. Berger, L. Valdevit, Plate-nanolattices at the theoretical limit of stiffness and strength. *Nat. Commun.* **11**, 1579 (2020).

50. Y. Wang, X. Zhang, Z. Li, H. Gao, X. Li, Achieving the theoretical limit of strength in shell-based carbon nanolattices. *Proc. Natl. Acad. Sci. U.S.A.* **119**, e2119536119 (2022).

51. X. Zhang, A. Vyatskikh, H. Gao, J. R. Greer, X. Li, Lightweight, flaw-tolerant, and ultrastrong nanoarchitected carbon. *Proc. Natl. Acad. Sci. U.S.A.* **116**, 6665–6672 (2019).

52. Y. Wang, K. Wu, X. Zhang, X. Li, Y. Wang, H. Gao, Superior fracture resistance and topology-induced intrinsic toughening mechanism in 3D shell-based lattice metamaterials. *Sci. Adv.* **10**, eadq2664 (2024).

53. M. Eisenstein, Seven technologies to watch in 2024. *Nature* **625**, 844–848 (2024).

54. J. Hu, Y. N. Shi, X. Sauvage, G. Sha, K. Lu, Grain boundary stability governs hardening and softening in extremely fine nanograined metals. *Science* **355**, 1292–1296 (2017).

55. A. van de Walle, P. Tiwary, M. de Jong, D. L. Olmsted, M. Asta, A. Dick, D. Shin, Y. Wang, L. Q. Chen, Z. K. Liu, Efficient stochastic generation of special quasirandom structures. *Calphad-Comput. Coupling Ph. Diagrams Thermochem.* **42**, 13–18 (2013).

56. S. Wang, J. Xiong, D. Li, Q. Zeng, M. Xiong, X. Chai, Comparison of two calculation models for high entropy alloys: Virtual crystal approximation and special quasi-random structure. *Mater. Lett.* **282**, 128754 (2021).

57. J. P. Perdew, K. Burke, M. Ernzerhof, Generalized gradient approximation made simple. *Phys. Rev. Lett.* **77**, 3865–3868 (1996).

58. G. Kresse, D. Joubert, From ultrasoft pseudopotentials to the projector augmented-wave method. *Phys. Rev. B* **59**, 1758–1775 (1999).

59. P. E. Blöchl, Projector augmented-wave method. *Phys. Rev. B* **50**, 17953–17979 (1994).

60. J. Lubliner, J. Oliver, S. Oller, E. Oñate, A plastic-damage model for concrete. *Int. J. Solids Struct.* **25**, 299–326 (1989).

61. Z. Du, C. Wu, Y. Chen, Q. Zhu, Y. Cui, H. Wang, Y. Zhang, X. Chen, J. Shang, B. Li, W. Chen, C. Liu, S. Yang, High-entropy carbonitride MAX phases and their derivative MXenes. *Adv. Energy Mater.* **12**, 202103228 (2021).

62. F. Bouville, E. Maire, S. Meille, B. Van de Moortèle, A. J. Stevenson, S. Deville, Strong, tough and stiff bioinspired ceramics from brittle constituents. *Nat. Mater.* **13**, 508–514 (2014).

63. L. J. Gibson, M. F. Ashby, *Cellular Solids: Structure and Properties* (Cambridge Univ. Press, 1999).

64. D. N. Leonard, G. W. Chandler, S. Seraphin, in *Characterization of Materials*, E. N. Kaufmann, Ed. (John Wiley & Sons Inc., 2012), vol. 2, pp. 1721–1735.

65. A. K. Geim, K. S. Novoselov, The rise of graphene. *Nat. Mater.* **6**, 183–191 (2007).

66. Y. Bai, R. Zhang, X. Ye, Z. Zhu, H. Xie, B. Shen, D. Cai, B. Liu, C. Zhang, Z. Jia, S. Zhang, X. Li, F. Wei, Carbon nanotube bundles with tensile strength over 80 GPa. *Nat. Nanotechnol.* **13**, 589–595 (2018).

67. A. King, G. Johnson, D. Engelberg, W. Ludwig, J. Marrow, Observations of intergranular stress corrosion cracking in a grain-mapped polycrystal. *Science* **321**, 382–385 (2008).

68. C. Dang, J.-P. Chou, B. Dai, C.-T. Chou, Y. Yang, R. Fan, W. Lin, F. Meng, A. Hu, J. Zhu, J. Han, A. M. Minor, J. Li, Y. Lu, Achieving large uniform tensile elasticity in microfabricated diamond. *Science* **371**, 76–78 (2021).

69. J. U. Surjadi, Y. Zhou, S. Huang, L. Wang, M. Li, S. Fan, X. Li, J. Zhou, R. H. W. Lam, Z. Wang, Y. Lu, Lightweight, ultra-tough, 3D-architected hybrid carbon microlattices. *Matter* **5**, 4029–4046 (2022).

70. S. Zhang, D. Chen, Z. Liu, M. Ruan, Z. Guo, Novel strategy for efficient water splitting through pyro-electric and pyro-photo-electric catalysis of $BaTiO_3$ by using thermal resource and solar energy. *Appl. Catal. B Environ.* **284**, 119686 (2021).

71. X. Xu, L. Xiao, Y. Jia, Z. Wu, F. Wang, Y. Wang, N. O. Haugen, H. Huang, Pyro-catalytic hydrogen evolution by $Ba_{0.7}Sr_{0.3}TiO_3$ nanoparticles: Harvesting cold–hot alternation energy near room-temperature. *Energy Environ. Sci.* **11**, 2198–2207 (2018).

72. Y. Pan, X. Xu, Y. Zhong, L. Ge, Y. Chen, J. M. Veder, D. Guan, R. O'Hayre, M. Li, G. Wang, H. Wang, W. Zhou, Z. Shao, Direct evidence of boosted oxygen evolution over perovskite by enhanced lattice oxygen participation. *Nat. Commun.* **11**, 2002 (2020).

73. T. X. Nguyen, Y. C. Liao, C. C. Lin, Y. H. Su, J. M. Ting, Advanced high entropy perovskite oxide electrocatalyst for oxygen evolution reaction. *Adv. Funct. Mater.* **31**, 202101632 (2021).

74. T. D. Thanh, N. D. Chuong, J. Balamurugan, H. Van Hien, N. H. Kim, J. H. Lee, Porous hollow-structured $LaNiO_3$ stabilized N,S-codoped graphene as an active electrocatalyst for oxygen reduction reaction. *Small* **13**, 201701884 (2017).

75. C. Li, K. C. K. Soh, P. Wu, Formability of ABO_3 perovskites. *J. Alloy. Compd.* **372**, 40–48 (2004).

76. H. Kim, R. Pingali, S. K. Saha, Rapid printing of nanoporous 3D structures by overcoming the proximity effects in projection two-photon lithography. *Virtual and Phys. Prototyp.* **18**, e2230979 (2023).

77. J. Bauer, A. Schroer, R. Schwaiger, O. Kraft, The impact of size and loading direction on the strength of architected lattice materials. *Adv. Eng. Mater.* **18**, 1537–1543 (2016).

78. J. J. do Rosário, J. B. Berger, E. T. Lilleodden, R. M. McMeeking, G. A. Schneider, The stiffness and strength of metamaterials based on the inverse opal architecture. *Extreme Mech. Lett.* **12**, 86–96 (2017).

79. J. Bauer, C. Crook, A. Guell Izard, Z. C. Eckel, N. Ruvalcaba, T. A. Schaedler, L. Valdevit, Additive manufacturing of ductile, ultrastrong polymer-derived nanoceramics. *Matter* **1**, 1547–1556 (2019).

80. X. Zheng, W. Smith, J. Jackson, B. Moran, H. Cui, D. Chen, J. Ye, N. Fang, N. Rodriguez, T. Weisgraber, C. M. Spadaccini, Multiscale metallic metamaterials. *Nat. Mater.* **15**, 1100–1106 (2016).

81. J. Bauer, A. Schroer, R. Schwaiger, O. Kraft, Approaching theoretical strength in glassy carbon nanolattices. *Nat. Mater.* **15**, 438–443 (2016).

82. X. Zhang, L. Zhong, A. Mateos, A. Kudo, A. Vyatskikh, H. Gao, J. R. Greer, X. Li, Theoretical strength and rubber-like behaviour in micro-sized pyrolytic carbon. *Nat. Nanotechnol.* **14**, 762–769 (2019).

83. J. Bauer, L. R. Meza, T. A. Schaedler, R. Schwaiger, X. Zheng, L. Valdevit, Nanolattices: An emerging class of mechanical metamaterials. *Adv. Mater.* **29**, 1701850 (2017).

84. A. J. Jacobsen, S. Mahoney, W. B. Carter, S. Nutt, Vitreous carbon micro-lattice structures. *Carbon* **49**, 1025–1032 (2011).

85. A. Guell Izard, J. Bauer, C. Crook, V. Turlo, L. Valdevit, Ultrahigh energy absorption multifunctional spinodal nanoarchitectures. *Small* **15**, 201903834 (2019).

86. A. Torrents, T. A. Schaedler, A. J. Jacobsen, W. B. Carter, L. Valdevit, Characterization of nickel-based microlattice materials with structural hierarchy from the nanometer to the millimeter scale. *Acta Mater.* **60**, 3511–3523 (2012).

87. M. Mieszala, M. Hasegawa, G. Guillonneau, J. Bauer, R. Raghavan, C. Frantz, O. Kraft, S. Mischler, J. Michler, L. Philippe, Micromechanics of amorphous metal/polymer hybrid structures with 3D cellular architectures: Size effects, buckling behavior, and energy absorption capability. *Small* **13**, 1602514 (2016).

88. L. Brigo, J. E. M. Schmidt, A. Gandin, N. Michieli, P. Colombo, G. Brusatin, 3D nanofabrication of SiOC ceramic structures. *Adv. Sci.* **5**, 1800937 (2018).

89. L. Guo, H. Xia, H. T. Fan, Y. L. Zhang, Q. D. Chen, T. Zhang, H. B. Sun, Femtosecond laser direct patterning of sensing materials toward flexible integration of micronanosensors. *Opt. Lett.* **35**, 1695–1697 (2010).
